# Seismic magnitude clustering is prevalent in field and laboratory catalogs

**DOI:** 10.1038/s41467-023-37782-5

**Published:** 2023-04-12

**Authors:** Q. Xiong, M. R. Brudzinski, D. Gossett, Q. Lin, J. C. Hampton

**Affiliations:** 1 Geomechanics and Damage Group (GeoD), Department of Civil and Environmental Engineering, University of Wisconsin-Madison, Madison, WI USA; 2 Department of Geology and Environmental Earth Science, Miami University, Oxford, OH USA; 3 State Key Laboratory of Petroleum Resources and Prospecting, China University of Petroleum, 102249 Beijing, China; 4 College of Petroleum Engineering, China University of Petroleum, Beijing, China

**Keywords:** Seismology, Geophysics, Natural hazards

## Abstract

Clustering of earthquake magnitudes is still actively debated, compared to well-established spatial and temporal clustering. Magnitude clustering is not currently implemented in earthquake forecasting but would be important if larger magnitude events are more likely to be followed by similar sized events. Here we show statistically significant magnitude clustering present in many different field and laboratory catalogs at a wide range of spatial scales (mm to 1000 km). It is universal in field catalogs across fault types and tectonic/induced settings, while laboratory results are unaffected by loading protocol or rock types and show temporal stability. The absence of clustering can be imposed by a global tensile stress, although clustering still occurs when isolating to triggered event pairs or spatial patches where shear stress dominates. Magnitude clustering is most prominent at short time and distance scales and modeling indicates >20% repeating magnitudes in some cases, implying it can help to narrow physical mechanisms for seismogenesis.

## Abstract (web summary)

Clustering of earthquake magnitudes is actively debated. Here, the authors show statistically significant magnitude clustering present in many different field and laboratory catalogs at a wide range of spatial scales (mm to 1000 km).

## Introduction

Clustering in time and space is a well-recognized feature of earthquakes, with prominent examples being spatial clustering of aftershocks around a mainshock and Omori–Utsu decay in the temporal productivity^1,2^. These patterns are consistent with universal scaling laws for the temporal and spatial patterns between successive earthquakes^3,4^. However, the existence of clustering in earthquake magnitudes is still a matter of active debate. Other than the power-law characterization of the frequency-magnitude distribution (Gutenberg-Richter law)^5^, magnitudes were thought to be independent until a set of studies reported magnitude correlations between sequential cataloged earthquakes^6–8^. However, these results could be influenced by catalog incompleteness, questioning the significance of the observed correlations^9,10^. If magnitude clustering does exist, it has practical applications in the form of short-term forecasting, particularly if larger magnitude events can be clustered in short time windows^11–14^. Typical forecasting approaches, such as the epidemic-type aftershock sequence (ETAS) approach, utilize a methodology for simulating seismicity with spatial and temporal clustering but without magnitude clustering^13,15,16^. Determining whether magnitude clustering exists is also paramount to understanding fault behavior considering proposed magnitude correlations appear to be more apparent when earthquakes occur close-in-time and space^17,18^. If magnitude clustering is a universal feature of seismic behavior, it provides a new opportunity to evaluate physical mechanisms for seismogenesis.

In this study, we evaluate the existence of magnitude clustering extensively in a variety of field and lab settings.

## Results

### Prior studies and the Southern California catalog

Much of the prior work on magnitude clustering has focused on the Southern California catalog (e.g., ref. ^19^). Prior work found the magnitude of a given event may depend on the magnitude of the previous event^20^, the correlations between consecutive magnitudes are restricted to recurrence times <30 min^6^, and the next earthquake tends to have a magnitude similar but smaller than the previous one18. However, the limited observation of magnitude correlations to short recurrence times suggested it may be a spurious effect due to short-term aftershock incompleteness (STAI), and that overall catalog incompleteness associated with seismic network density increased the probability for the magnitudes of subsequent earthquakes to be similar^10^.

**Fig. 1:**
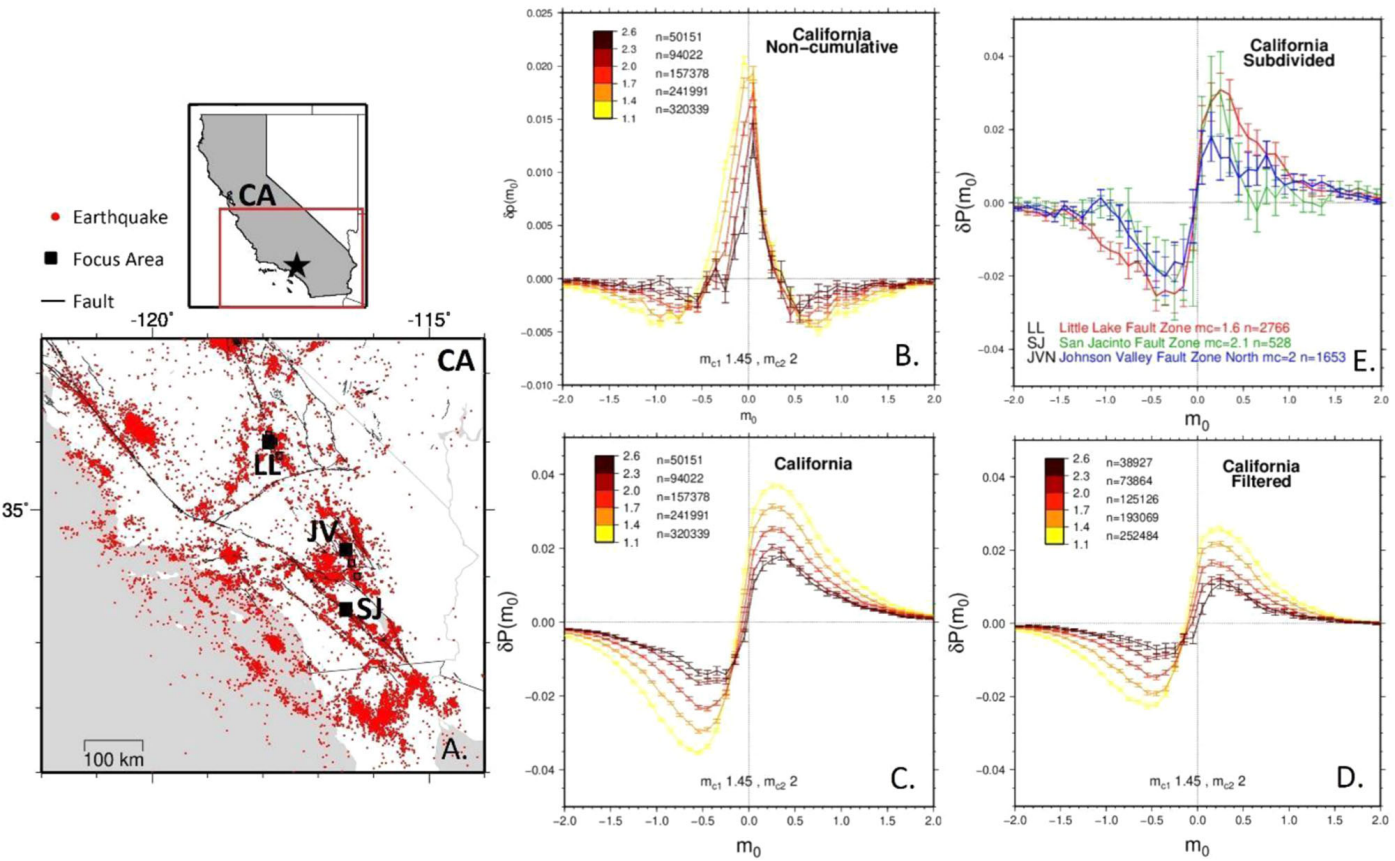
Study area and magnitude clustering plots. **A** Map of southern California study area. **B** Non-cumulative distribution of difference in probability between the observed catalog and a randomized version, $\delta P\left({m}_{0}\right)$, as a function of magnitude difference (${m}_{0}$), for the southern California catalog. ${m}_{c}=$ magnitude of completeness, *n* = number of events. Error bars correspond to the 1 standard deviation confidence interval. **C** Cumulative distribution of the difference in probability, $\delta P\left({m}_{0}\right)$, for the southern California catalog before applying filters. **D** Same as **C** but after applying filters to address potential issues from catalog incompleteness. **E** Cumulative distribution for 3 areas of 10×10 km^2^ in southern California with pronounced seismicity, represented by solid black boxes in the southern California map.

To address this debate, we performed a similar analysis on the same high precision Southern California catalog from 1985 to 2001 with more than 400,000 events^19^, (Fig. 1A). To test for magnitude clustering, we followed the approach of prior work in comparing the probabilistic distribution (*P*) of magnitude differences ($\triangle m$) of successive events between the real catalog and a randomly shuffled catalog using the equation $\delta p\left({m}_{0}\right)=P\left(\triangle m=\,{m}_{0}\right)-P(\triangle {m}^{*}=\,{m}_{0})$, where $\triangle {m}^{*}$ represents the magnitude differences after randomly shuffling the order of the cataloged events. If magnitude clustering is present, $\delta p\left({m}_{0}\right)$ should significantly deviate from zero for a given magnitude difference ${m}_{0}$. In a catalog with randomly arranged magnitudes, $\delta p\left({m}_{0}\right)$ would not deviate from zero. Our findings show that these statistically significant deviations do indeed occur, and they occur at several different magnitude thresholds (Fig. 1B). The largest probability occurs at the small magnitude differences. These deviations were most strongly observed in the cumulative distribution of magnitude differences: $\delta P\left({m}_{0}\right)=P\left(\triangle m < \,{m}_{0}\right)-P(\triangle {m}^{*} < \,{m}_{0})$ (Fig. 1C), which were the specific target of Davidsen and Green^10^.

### Demonstrating magnitude clustering despite catalog incompleteness

We investigated Davidsen and Green’s^10^ claims that magnitude incompleteness caused the apparent magnitude clustering by applying two standard approaches to estimate the magnitude of completeness via the frequency-magnitude distribution of the catalog^21^ (Fig. S1). The maximum curvature method generally produces a lower, more inclusive estimate^22^, while the b-value stability method produces a higher, more conservative estimate^23,24^. We also followed the Davidsen and Green^10^ approach to correct for STAI by removing magnitude differences during periods following larger mainshocks and excluding all event pairs separated by less than 2 min to address smaller mainshocks. Fig. 1D shows the signature of the magnitude clustering remains prominent even when strategies for addressing incompleteness are implemented. We also investigated the claim that spatial variability of the magnitude of completeness could contribute to magnitude clustering by focusing on smaller (10×10 km^2^) areas of the California catalog with the most productive seismicity. Although the uncertainties are larger for the smaller datasets, the magnitude clustering patterns remain statistically significant at these spatial scales along several different faults (Figs. 1E and S2). These findings are similar to Lippiello et al.^17^ that used two regions with different magnitude of completeness thresholds to argue that magnitude correlations do not depend on catalog incompleteness.

**Fig. 2:**
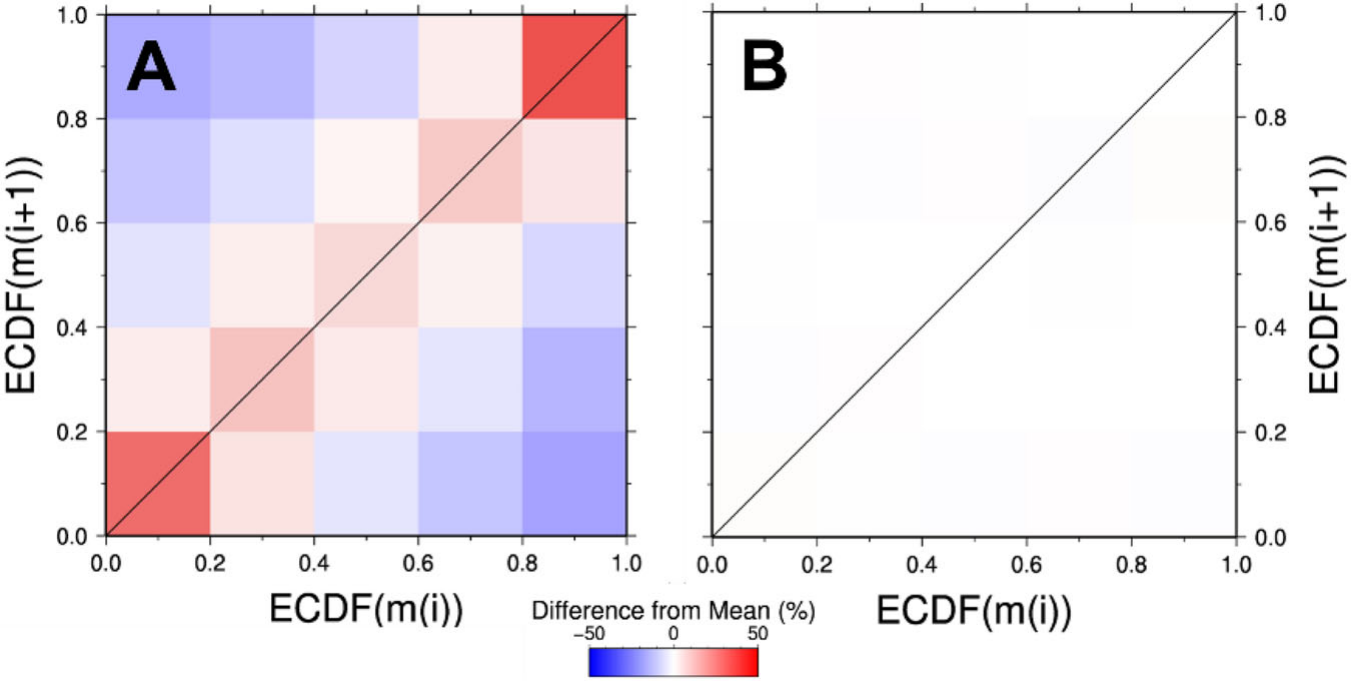
Empirical cumulative density function (ECDF) value of an event magnitude (m(i)) compared to that of the subsequent event (m(i + 1)). Comparisons are for **A** the real, filtered southern California catalog and **B** this catalog randomized by event time. Color scale shows the number of event comparisons that fall into each 0.2 × 0.2 bin by illustrating the difference relative to the expected mean (total number of comparisons divided by total number of bins). Diagonal line highlights cases where an event has the same ECDF bin value as the subsequent event.

To further demonstrate the statistical significance of magnitude clustering in spite of potential incompleteness that could proportionally influence smaller magnitude events, we developed a new approach to compare successive events based on their positions in the empirical cumulative density function (ECDF) of the magnitudes (see “Methods”). Figure 2A shows the results from calculating the number of event comparisons that fall into each bin. To help establish true variations from the catalog magnitude distribution, ECDF values were also calculated on the catalog randomized by time (Fig. 2B). Subsequent events with the same ECDF bin value (diagonal line) occurred at significantly higher rates than randomized catalogs. The biggest difference (+26%) occurred for the largest magnitude bin, highlighting that magnitude clustering is not restricted to small magnitude comparisons. We found a >99.9999% confidence level that the increased number of events along the 1:1 diagonal line for the real catalog were statistically different from that of 1000 randomly shuffled realizations of the catalog (Table S1).

### Application to Injection Induced Seismicity Catalogs

**Fig. 3:**
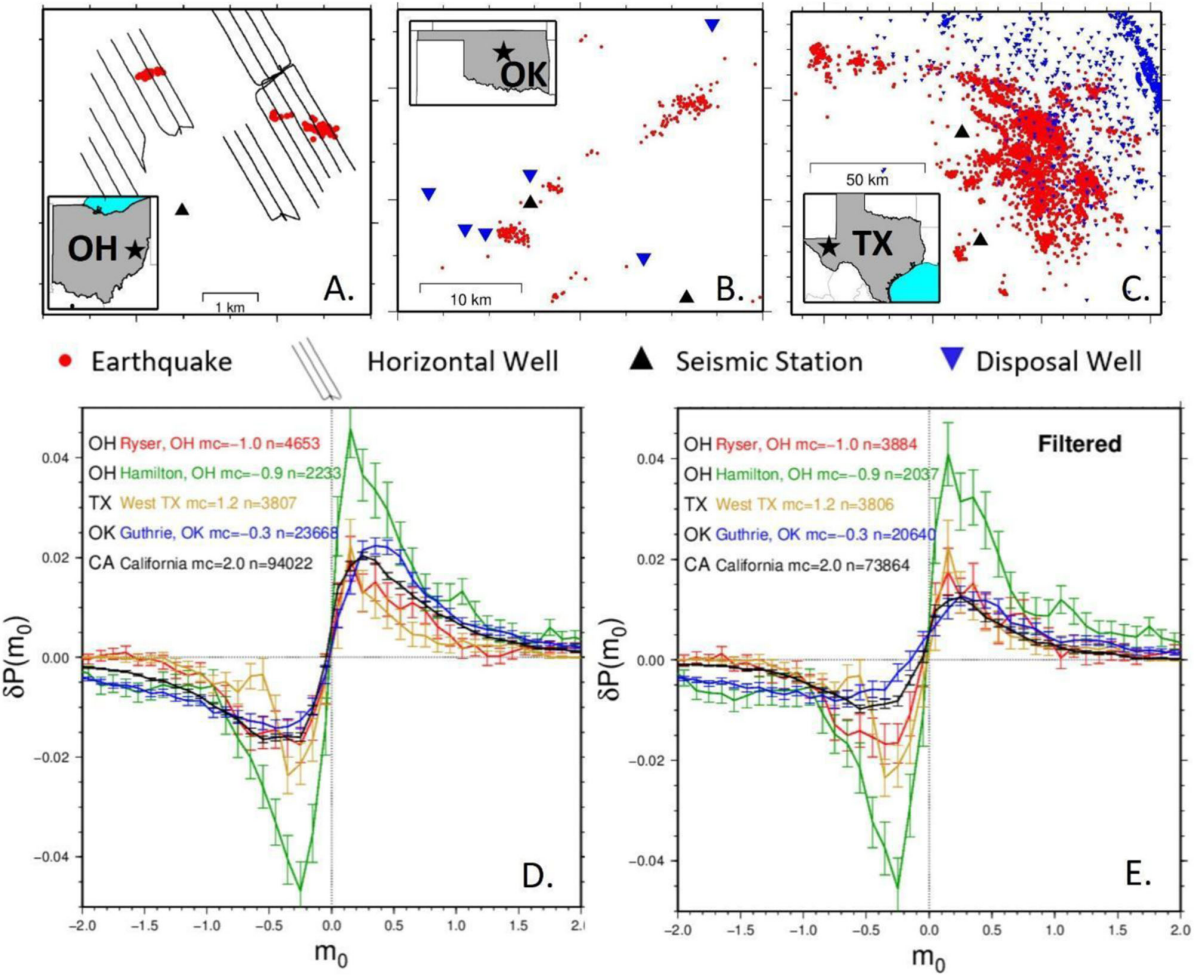
Study area and magnitude clustering plots. **A** Map of Harrison County, Ohio study area. Seismicity on the left side of the map is associated with the Ryser well pad, and seismicity on the right associated with Hamilton well pad. **B** Map of the Logan County, Oklahoma study area near the town of Guthrie. **C** Map of the west Texas study area. **D** Comparison of the cumulative distribution of difference in probability between the observed catalog and a randomized version, $\delta P\left({m}_{0}\right)$, as a function of magnitude difference (${m}_{0}$), for each catalog before any filters are applied. **E** Same as **D** but after applying filters to address potential issues from catalog incompleteness. Same labeling conventions as Fig. 1.

Although many of the studies investigating magnitude clustering have focused on California, some have not^9,11,12^. A noteworthy study is that of hydraulic fracturing induced seismicity that identified prominent magnitude clustering and interpreted it as a consequence of the specific geometrical constraints of finely laminated shale gas and tight oil reservoirs^25^. To investigate this idea, we turned our attention to a variety of field-scale human induced seismicity catalogs (Fig. 3). Two catalogs are from hydraulic fracturing well pads a few km apart in Harrison County, Ohio (Ryser and Hamilton)^26,27^. The other two catalogs are from wastewater disposal cases near Guthrie in central Oklahoma and the Delaware Basin in west Texas, which are about 10 km and 50 km wide, respectively^28–30^.

We find that all of these seismicity catalogs have significant magnitude clustering, with several nearly identical to results from the California catalog. Fig. 3D shows this after limiting to the conservative magnitude of completeness threshold, but the significance can be seen at even higher magnitude thresholds (Figs. S3 and S5). Perhaps the most noteworthy difference is the larger signature from the Hamilton catalog, which is intriguing, considering the similar conditions to the nearby Ryser catalog^26,27^, although Hamilton was stimulated 2 years after Ryser. While most of the catalogs in this comparison are from strike-slip environments (California, Ohio, Oklahoma), the West Texas catalog is dominated by normal faulting^31^, demonstrating that magnitude clustering is not restricted to a particular fault type at the field scale.

A key advantage of induced sequences is that they are swarms instead of aftershock sequences^32,33^, so it removes the concern about STAI when evaluating magnitude clustering. The induced sequences show the same degree of magnitude clustering as the California tectonic seismicity catalog (Fig. 3D), further supporting the notion that STAI is not artificially causing magnitude clustering observations^17,18^.

### Time dependency of magnitude clustering

**Fig. 4:**
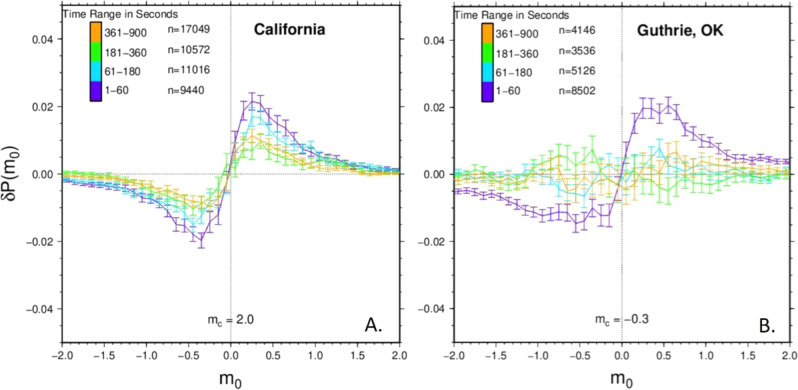
Comparison of A) California and B) Guthrie cumulative distribution after splitting the filtered versions of these catalogs into different time ranges. *n* = number of events, ${m}_{c}$ = magnitude of completeness. The filtered Guthrie catalog shows a decay in the magnitude clustering signature as the events are further separated in time.

The Guthrie catalog is noteworthy for its high seismicity rate^28–30^, and we determined that a shorter 10-s interevent time filter was still appropriate for this catalog given the advanced subspace detection technique. Even after implementing this shorter interevent time restriction, the size of the magnitude clustering signature was reduced by more than half in the Guthrie catalog (cf. Fig. 3D, E), indicating that magnitude clustering in this case is prominent among events with time separations less than 10 s. To further explore the effect of interevent time on magnitude clustering, we split each catalog into interevent time intervals (Fig. S11). Figure 4 shows how the Guthrie, Oklahoma wastewater disposal catalog has significant variation in magnitude clustering with different interevent times while the California tectonic catalog does not. This result provides intriguing clues that something about the wastewater disposal process is enabling magnitude clustering over shorter time scales but disrupting it over longer time scales.

### Application to laboratory catalogs

**Fig. 5:**
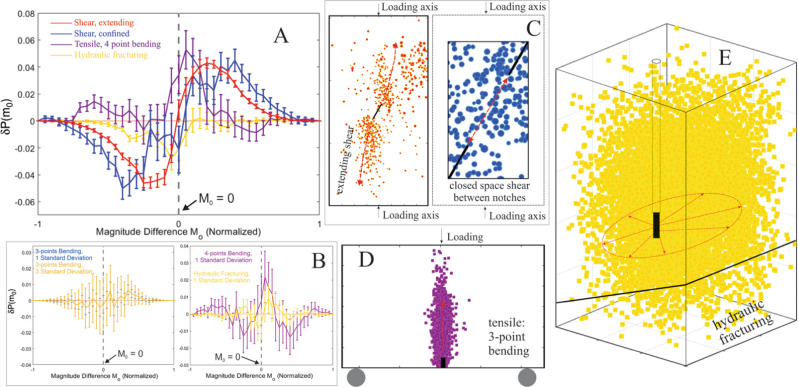
Magnitude clustering in laboratory earthquakes. **A** Normalized magnitude clustering phenomena for various types of rock fractures on different rock types. Absolute *M*_0_ spans of the curves are varied as tests are conducted on different rock types with different data acquisition systems. **B** The non-clustering observations on the whole catalogs of the rock fractures under dominatingly tensile stress. Catalogs are on tests conducted at University of Minnesota (left), and Halliburton (right), respectively. **C**–**E** The induced rock fractures under extending and shear stress^35^ conditions (left of **C**), confined and shear stress^39^ conditions (right of **C**), tensile stress^42,43^ conditions (**D**), and hydraulic fracturing (**E**)^40,41^.

Based on the verification of magnitude clustering in a variety of field environments, we turned our attention to the laboratory environment to see whether these patterns would persist at even smaller scales and to probe the physical mechanisms. The wider variability and controlled conditions of environments in the laboratory provides an opportunity to explore the necessary conditions and the potential controlling factors for magnitude clustering. The fundamental similarities between laboratory rock fracture processes and seismogenic processes are well documented^34–37^. The investigated tests cover more than a decade of effort of different academic and industrial research laboratories, but all the laboratory catalogs display scale-invariance features in that they obey Gutenberg–Richter magnitude–frequency power–law and even can exhibit universal scaling laws for interevent times and distances^35,38–44^. The lab catalog spans of absolute magnitude difference (*M*_0_) are variable due to the differences of rock types and data acquisition systems used at different industrial and research institutes but are generally smaller than the field studies. These tests also cover a wide range of different loading protocols and stress conditions in multiple rock types (see “Methods”), so we found it useful to divide into those generating rock fracture under shear stress, either under extending^35^ or confined^39^ conditions (Fig. 5C), and those causing rock fractures under dominatingly tensile stress, including tensile bending and hydraulic fracturing^40–43^ (Fig. 5D, E).

### Magnitude clustering in laboratory catalogs

We found magnitude clustering in rock fracture under shear stress occurs regardless of loading protocols, rock types, and observable magnitude ranges imposed by different data acquisition systems. As in the field-scale investigations, we addressed potential issues of catalog incompleteness in our processing of the laboratory catalogs. Figure 5a illustrates how prominent magnitude clustering occurs under both extending^35^ and confined^39^ shear rock fractures. Significant magnitude clustering occurs regardless of whether the magnitude of completeness is met (Figs. S12a and S15b). In fact, magnitude clustering appears to be “unconditional” in shear rock fracture tests as it can be observed regardless of temporal, spatial, loading protocol, or magnitude conditioning (Supplementary Note 2). The lack of influence of spatial condition indicates geometric constraints are not a first order control for magnitude clustering under shear stress. The energy input for these tests is also not the artificial factor for imposing magnitude clustering, as the energy input for most of the tests is progressively decreasing during the fracture processes, and unconditional clustering for shear rock fractures can be observed under both increasing and decreasing energy input conditions (Figs. S12, S16, and S17). The clustering became more significant as we progressively removed the AE events of the early testing time from the analysis and kept only the last several-hundred events (Fig. S14a). Such observation suggests the rock fracture evolution can amplify the magnitude clustering phenomena in shear rock fractures, but this influence is secondary as it did not alter the statistical significance of magnitude clustering.

### Effects of stress condition and interevent distance

For rock fracture under dominatingly tensile stress (tensile bending and hydraulic fracturing), the overall catalogs did not show statistically significant magnitude clustering (Fig. 5B). However, we were able to find significant magnitude clustering in these catalogs when we restricted the interevent distance (Figs. 5A and S12b). Specifically, the non-clustering pattern changed to significant clustering when the inter-event distance was conditioned to the range of the tests’ characteristic length (i.e., the influence of geometric constraints). For the hydraulic fracturing test this characteristic length is the distance between the fracturing wellbore and the pre-cut fault (Fig. S13), and for the tensile bending tests this length is the thickness of the specimens (Fig. S12b). The increase of statistical significance in magnitude clustering are also most clear when interevent distance conditioning approaches the characteristic length of the specimen. Some clues to why this restriction is necessary to observed magnitude clustering come from additional observations during tests that generated both tensile and shear rock fracture in the same sample^38^ (Fig. S17). Non-clustering occurred when the wing-shaped rock fractures were developing under tensile stress, but the later developing shear fracture shifted the magnitude clustering behavior to significant clustering. This indicates the remarkable finding that significant (i.e., exceeding 3-standard deviations) magnitude clustering appears to be universal when a shear rock fracture condition is met.

### Lack of magnitude clustering in synthetic catalogs

**Fig. 6:**
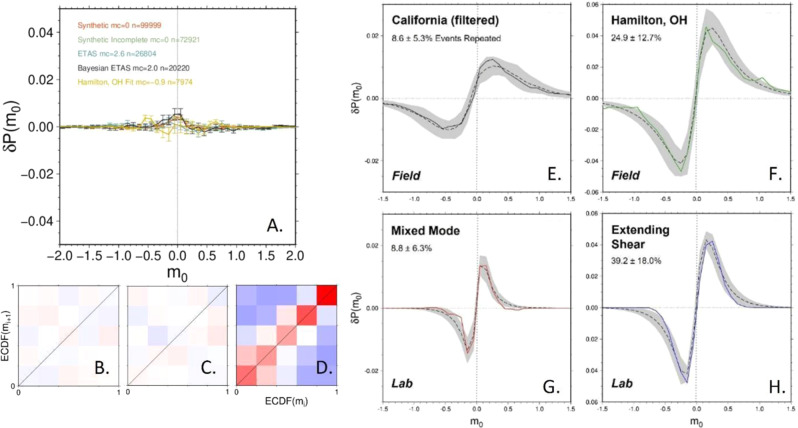
Key results of synthetic catalog analysis. **A** Comparison of cumulative distribution for synthetic catalogs generated with different approaches, including adding artificial incompleteness to the stochastic catalog and tuning the ETAS parameters to the Hamilton, OH catalog. ECDF results for the **B** ETAS catalog fit to Hamilton parameters, **C** stochastic catalog with incompleteness added, and **D** 22% repeating events added to the ETAS catalog. Magnitude clustering patterns observed in field (**E**, **F**) and lab (**G**, **H**) settings compared with best-fitting patterns from synthetic seismicity catalogs with varying amounts of repetitive events. A grid search over repeating percentage, magnitude uncertainty, and b-value was used to identify a range of synthetic catalogs that best fit the observed pattern (gray shading, dashed line indicates mean). Percentages of repeating events for the range of fitting catalogs are reported.

We investigated whether magnitude clustering exists in synthetics catalogs generated with a variety of techniques to identify whether magnitude clustering arises from existing knowledge of earthquake patterns (see “Methods”). None of these approaches, including several ETAS strategies and random draws from a FMD^45–47^, produced any statistically significant signature of magnitude clustering (Figs. 6 and S10). This includes when catalog incompleteness is artificially included or when the ETAS parameters are tuned to earthquake catalogs with magnitude clustering in them. These findings indicate that current strategies for modeling earthquake magnitudes, and the forecasting strategies that result, are unable to account for the temporal-based magnitude relationships demonstrated in this study.

## Discussion

### Modeling of observations catalogs with repeating events added to synthetic catalogs

To help investigate the causes of the magnitude clustering patterns demonstrated in multiple field and laboratory environments, one possibility to explain the observed patterns of magnitude clustering is if a higher than expected portion of events in the catalog are repeated events with approximately the same magnitude. We generated synthetic seismic catalogs and added repeating events to look for similarities to observed catalogs (see “Methods”). Figure 6 shows the best-fitting curves and shaded 2σ from the synthetic catalogs compared with a pair of field and lab catalogs, along with the corresponding percentage of inserted repeating events. For the California and mixed mode catalogs, a modest amount (~10%) of the event magnitudes repetition is needed to fit the data, although the uncertainty from this approach indicates it could be as small as 2%. For the Hamilton and extending shear catalogs, a larger portion (25–40%) of event magnitudes repetition, with at least 12–21% repeating based on the 2*σ*. The ideal laboratory environment under shear dominant stress is best fit by including 39% repeating events. Even though insertion of repeated events into synthetically generated catalogs is a simplistic way to envision the true process, it indicates a substantial relationship between magnitudes in observed seismicity catalogs.

### Potential physical mechanisms for magnitude clustering

We then used the laboratory catalogs to evaluate the potential physical mechanisms for magnitude clustering. Hypotheses we sought to evaluate were (1) whether fault patches rupture with incomplete strain release such that they can rupture again soon after to form similar size events or (2) whether there are conditions controlling event size that change slowly enough between events to produce a clustering of magnitudes. The precision of the lab catalogs allowed us to define triggered pairs (one event triggers an aftershock) based on whether they violate the null hypothesis that events occur randomly in space, time, and magnitude following well-established scaling relationships^35,48–52^ (see Supplementary Information).

### Influence of triggering

**Fig. 7:**
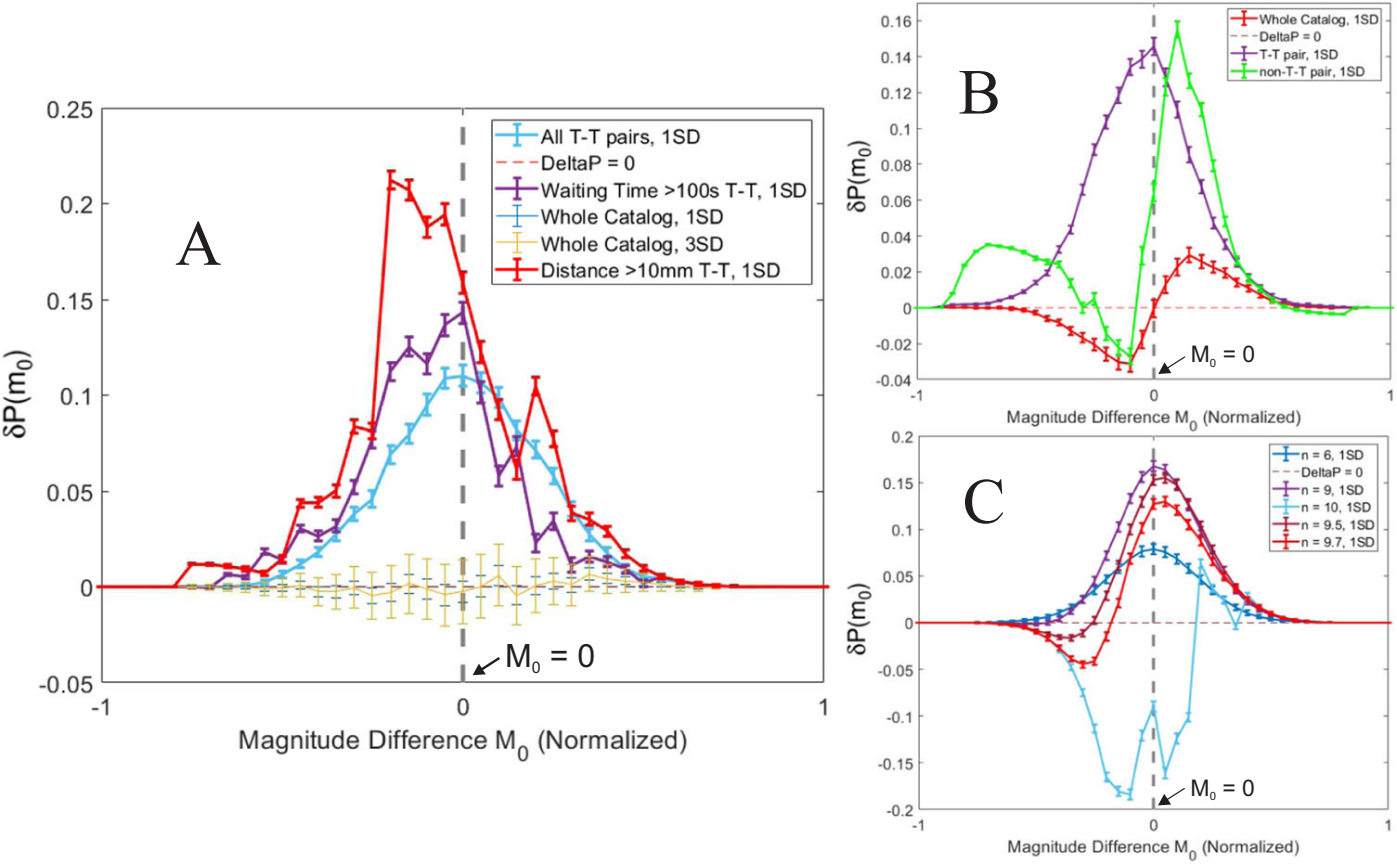
Cumulative distributions of difference in probability between portions of observed catalogs and a randomized version of the full catalog. **A** Magnitude clustering of all triggering-triggered (T-T) pairs (cyan), long T-T waiting time pairs (purple), long T-T distance pairs (red), derived from a tensile stress catalog that did not show significant magnitude clustering (whole catalog, 1 or 3 SD, yellow)^42,43^. **B** Different magnitude clustering patterns of T-T or non-T-T pairs, derived from extending and shear stress^35^ conditions. **C** Shifting of magnitude clustering pattern by removing all possible pairs below a threshold (*n*) in the normalized space-and-time distance, derived from tensile stress^42,43^ conditions.

Intriguingly, restricting to triggering-triggered (T-T) pairs created the most significant magnitude clustering patterns we observed in rock fracture under dominatingly tensile stress, far exceeding the increases under all other types of interevent spatial and/or temporal constraints discussed above (Fig. 7A).

The magnitude clustering pattern for the T-T pairs shows significantly higher probability for triggered events to be smaller magnitude (higher probability in negative M_0_ range). We have elucidated in previous research that, observing a smaller magnitude event is expectable for remotely triggered events^35^. Narrowing our focus to long distance (>10 mm) T-T pairs or long waiting time (<100 s) T-T pairs produce magnitude clustering patterns of even higher significance (Fig. 7A). Moreover, once we remove all event pairs violating the null hypothesis of GR law and preserve the pairs obeying that null hypothesis, we can even observe distinctively different types of significant magnitude clustering (Fig. 7B, C), i.e., significantly lower probability in negative *M*_0_ range (Fig. 7C). Such observation suggests a possibility that, the overlapping of different magnitude clustering patterns from obeying or violating the null hypothesis of GR law in different ratios can result in globally non-clustering or clustering observations.

### Future work

The discovery that magnitude clustering is pervasive in both lab and field seismicity but with different prevalence depending on specific conditions provides tantalizing opportunities to explore physical mechanisms with future work. Hypotheses to evaluate would include (1) whether fault patches rupture with incomplete strain release such that they can rupture again soon after to form similar size events or (2) whether there are conditions controlling event size that change slowly enough between events to produce a clustering of magnitudes. For example, the increased magnitude clustering signature for long distance T-T pairs appears to be inconsistent with the identical patch rupture hypothesis, unless the incomplete strain release leads to transferred stress that promotes slip on patches of similar but smaller strength. Regardless, improving characterizations of magnitude clustering that integrate laboratory and field scale observations will narrow the possible physical mechanisms for earthquakes.

## Methods

### Data sources

Seismicity associated with the Ryser well pad was locally recorded between September 2013 and January 2014. Seismicity associated with the Hamilton well pad was locally recorded between August and November 2015. The seismicity catalogs were enhanced using a repeating signal detection algorithm based on waveform similarity^26^. Wells on the Ryser pad were stimulated sequentially (one lateral at a time), while operations on the Hamilton pad utilized a common “zipper-frac” approach where stages are alternated between two well laterals^27^. The Guthrie seismicity catalog was locally recorded in Logan County, Oklahoma between February and August 2014. The catalog was enhanced using a subspace detection technique^28^. It is well established that seismicity in central Oklahoma during this time was induced by widespread, large rate wastewater disposal^53^. The West Texas seismicity catalog was regionally recorded between March 2017 and December 2018^29^. The catalog was enhanced using regional template matching^30^.

The investigated laboratory tests include a variety of different types of laboratory tests (3- and 4-point bending tensile tests, flawed rock compressive tests, hydraulic fracturing test; with different loading paths or protocols) under different types of stress conditions (tensile, shear, and hydraulic fracturing stress conditions) on a variety of different types of rocks (Dakota Granite, Carrara Marble, Berea sandstone, and sand-mixed cement, with different levels of homogeneity). These tests were conducted in different laboratories at different institutes (University of Minnesota-Twin Cities, Colorado School of Mines, Rock Mechanics Lab of Halliburton, and Nanyang Technological University, Singapore) spanning a decade. The energy releases from the rock fracture processes are also recorded by different types of acquisition systems. See supplementary Information for more details on specific tests.

### The cumulative distribution of the probability comparison

The cumulative distribution of the difference in probability between the observed catalog and a randomized version as a function of magnitude difference shows a sinusoidal pattern for our observational catalogs. During the larger negative x-values (${m}_{0}$), the randomized catalogs have a larger probability of producing this larger negative ${m}_{0}$, because the real catalog is deficient in larger negative ${m}_{0}$ values as it tends to have more smaller ${m}_{0}$ values (both negative and positive). In essence, the real catalog is "trailing behind" the randomized catalogs in terms of cumulative percentage of events when we are on the negative part of the ${m}_{0}$ axis. The real catalog flips to being ahead of the randomized catalog once we enter the positive part of the ${m}_{0}$ axis as all of the small ${m}_{0}$ values allow it to surpass the relative percentage of the randomized catalogs. This pattern is not seen in Fig. 7, because only a portion of the observed catalog is being compared to a randomized version of the full catalog, such that the comparisons do not sum to the same number of events in a cumulative distribution. The consistently positive values occur due to the higher probability of observing similar magnitude events through the triggering-triggered (T-T) pairs.

### Empirical cumulative density function for magnitudes

The ECDF was calculated by sorting the already filtered catalog magnitudes from smallest to largest and assigning a value equal to the count divided by the total number of events. The catalog was then resorted by time and the ECDF value of each event (i) was compared to the ECDF value of the subsequent event (*i* + 1).

### Determining the time filter to address interevent incompleteness

Following the approach of prior research^10^, we estimated the temporary completeness magnitude *m*_tc_(*t*,*m*) at time *t* (in days) after a mainshock of magnitude *m*: *m*_tc_(*t*,*m*) = *m* − 4.5 − 0.75 log_10_(*t*). Thus we corrected for STAI by removing earthquakes and their magnitude differences during periods where *m*_tc_ (*t*,*m*) > *m*_c_ for all mainshocks with *m* ≥ 6. For the filtering to avoid events hidden within coda of recent event, we used a minimum interevent time of 2 min for the California catalog as in prior work^10^, However, when we examined seismograms associated with our induced seismicity catalogs, we identified that this time filter could be reduced to at least 30 seconds given the amplitude of small events and rates of coda decay.

### Synthetic catalog processing

The stochastic synthetic catalogs were drawn from a probability density function based on the Gutenberg-Richter magnitude-frequency relation (e.g., Zhuang and Touati^45^). We explored issues with catalog incompleteness by artificially removing an increasing number of events towards the smaller magnitude end of the catalog. We also investigated issues with magnitude uncertainty by smearing via a normal distribution using the Box-Muller Transform. When seeking to model the observed catalogs, we generated stochastic catalogs for a range of different b-values, magnitude uncertainties, and percentage of events that were repeated. Goodness of fit to cumulative magnitude clustering curves between the real catalog and synthetic catalogs from grid search of the parameters were evaluated using a probability density function for the chi-squared statistic. We also used two different methods of simulating catalogs based on the ETAS approach that incorporate aftershock triggering. The ETAS model incorporates the Gutenberg–Richter Law, the Omori-Utsu Decay Law, and spatial clustering of aftershocks to assign an occurrence rate ${R}_{0}(m,x,y,t)$ of events with magnitudes $m > \,{m}_{0}$ at a position $(x,y)$ during time $t$. The first ETAS method used estimates the ETAS parameters based on the expectation maximization (EM) algorithm originally applied to the ETAS model by Veen and Schoenberg^54^ in 2008^47^. The important parameters for the model are the background rate *μ* and the parameters *k*_0_, *α*, *c*, *ω*, *τ*, *d*, γ, *ρ*, which define the aftershock triggering rate ${R}_{0}$. We used the default estimates of the parameters.

We also applied a Bayesian approach to ETAS simulation that incorporates a maximum likelihood estimation to determine the model parameters^46^. Rather than using individual point estimates for the model parameters, it draws upon the posterior distribution $p(\theta {|Y})$, where $\theta$ equals an unknown parameter vector. This posterior distribution represents uncertainty in $\theta$ based on observed catalogs and any prior knowledge from previous studies, which can then be incorporated into the ETAS modeling by averaging the forecast distribution over the posterior. Initially, we simulated an ETAS catalog of ~20,000 events using the default parameters. We also used the maximum likelihood estimation to fit the model parameters using the observed data in both the Southern California Catalog and the Hamilton, OH catalogs, and then created simulated catalogs using these parameters.

## Supplementary information

Supplementary Information

## Data Availability

The field, laboratory, and synthetic catalogs are available via 10.5281/zenodo.7328585^55^.

## Code availability

Codes for analyses can be available upon request by reaching out to Professor Jesse Hampton at University of Wisconsin-Madison (jesse.hampton@wisc.edu).

## References

[1] Utsu T, Ogata Y, Matsu’ura RS (1995). The centenary of the Omori formula for a decay law of aftershock activity. J. Phys. Earth.

[2] Felzer KR, Brodsky EE (2006). Decay of aftershock density with distance indicates triggering by dynamic stress. Nature.

[3] Saichev A, Sornette D (2006). “Universal” distribution of interearthquake times explained. Phys. Rev. Lett..

[4] Davidsen J, Paczuski M (2005). Analysis of the spatial distribution between successive earthquakes. Phys. Rev. Lett..

[5] Gutenberg B, Richter CF (1944). Frequency of earthquakes in California. Bull. Seismol. Soc. Am..

[6] Corral Á (2006). Dependence of earthquake recurrence times and independence of magnitudes on seismicity history. Tectonophysics.

[7] Corral A (2004). Long-term clustering, scaling, and universality in the temporal occurrence of earthquakes. Phys. Rev. Lett..

[8] Lippiello E, Godano C, de Arcangelis L (2007). Dynamical scaling in branching models for seismicity. Phys. Rev. Lett..

[9] Davidsen J, Kwiatek G, Dresen G (2012). No evidence of magnitude clustering in an aftershock sequence of nano- and picoseismicity. Phys. Rev. Lett..

[10] Davidsen J, Green A (2011). Are earthquake magnitudes clustered?. Phys. Rev. Lett..

[11] Nichols K, Schoenberg FP (2014). Assessing the dependency between the magnitudes of earthquakes and the magnitudes of their aftershocks. Environmetrics.

[12] Spassiani I, Sebastiani G (2016). Exploring the relationship between the magnitudes of seismic events. J. Geophys. Res. Solid Earth.

[13] Field EH (2017). A spatiotemporal clustering model for the third uniform California earthquake rupture forecast (UCERF3‐ETAS): toward an operational earthquake forecast. Bull. Seismol. Soc. Am..

[14] Nandan S, Ouillon G, Sornette D (2019). Magnitude of earthquakes controls the size distribution of their triggered events. J. Geophys. Res. Solid Earth.

[15] Ogata YJ (1988). Statistical models of point occurrences and residual analysis for point processes. J. Am. Stat. Assoc..

[16] Hardebeck, J. L. *Appendix S: Constraining Epidemic Type Aftershock Sequence (ETAS) Parameters from the Uniform California Earthquake Rupture Forecast, Version 3 Catalog and Validating the ETAS Model for Magnitude 6.5 or Greater Earthquakes*. Report No. Open-File Report 2013-1165-S, and California Geological Survey Special Report 228-S (U.S. Geological Survey).(2013)

[17] Lippiello E, Godano C, de Arcangelis L (2012). The earthquake magnitude is influenced by previous seismicity. Geophys. Res. Lett..

[18] Lippiello E, de Arcangelis L, Godano C (2008). Influence of time and space correlations on earthquake magnitude. Phys. Rev. Lett..

[19] Hauksson E, Yang W, Shearer PM (2012). Waveform relocated earthquake catalog for Southern California (1981 to June 2011. Bull. Seismol. Soc. Am..

[20] Corral A (2005). Comment on “Do earthquakes exhibit self-organized criticality”. Phys. Rev. Lett..

[21] Mignan, A. & Woessner, J. Estimating the magnitude of completeness for earthquake catalogs. *Commun. Online Resourc. Stat. Seismicity Anal.*10.5078/corssa-00180805 (2012).

[22] Wiemer S, Wyss M (2000). Minimum magnitude of completeness in earthquake catalogs, examples from Alaska, the Western United States, and Japan. Bull. Seismol. Soc. Am..

[23] Cao A, Gao SS (2002). Temporal variation of seismicb-values beneath northeastern Japan island arc. Geophys. Res. Lett..

[24] Woessner J, Wiemer S (2005). Assessing the quality of earthquake catalogues: estimating the magnitude of completeness and its uncertainty. Bull. Seismol. Soc. Am..

[25] Maghsoudi S, Eaton DW, Davidsen J (2016). Nontrivial clustering of microseismicity induced by hydraulic fracturing. Geophys. Res. Lett..

[26] Skoumal RJ, Brudzinski MR, Currie BS (2016). An efficient repeating signal detector to investigate earthquake swarms. J. Geophys. Res. Solid Earth.

[27] Kozłowska M (2018). Maturity of nearby faults influences seismic hazard from hydraulic fracturing. Proc. Natl Acad. Sci. USA.

[28] Benz HM, McMahon ND, Aster RC, McNamara DE, Harris DB (2015). Hundreds of earthquakes per day: the 2014 Guthrie, Oklahoma, Earthquake Sequence. Seismol. Res. Lett..

[29] Savvaidis, A., Young, B., Huang, G. C. D. & Lomax, A. TexNet: a statewide seismological network in Texas. *Seismol. Res. Lett.*10.1785/0220180350 (2019).

[30] Skoumal, R. J., Barbour, A. J., Brudzinski, M. R., Langenkamp, T. & Kaven, J. O. Induced seismicity in the Delaware Basin, Texas. *J. Geophys. Res. Solid Earth*10.1029/2019jb018558 (2020).

[31] Snee J-EL, Zoback MD (2018). State of stress in the Permian Basin, Texas and New Mexico: implications for induced seismicity. Lead. Edge.

[32] Skoumal RJ, Brudzinski MR, Currie BS (2015). Distinguishing induced seismicity from natural seismicity in Ohio: Demonstrating the utility of waveform template matching. J. Geophys. Res. Solid Earth.

[33] Schultz, R. et al. Hydraulic fracturing‐induced seismicity. *Rev. Geophys.*10.1029/2019rg000695 (2020).

[34] Lockner DA, Byerlee JD, Kuksenko V, Ponomarev A, Sidorin A (1991). Quasi-static fault growth and shear fracture energy in granite. Nature.

[35] Xiong, Q. & Hampton, J. C. Non-local triggering in rock fracture. *J. Geophys. Res. Solid Earth*10.1029/2020JB020403 (2020).

[36] Reches Z (1999). Mechanisms of slip nucleation during earthquakes. Earth Planet. Sci. Lett..

[37] Bares J, Dubois A, Hattali L, Dalmas D, Bonamy D (2018). Aftershock sequences and seismic-like organization of acoustic events produced by a single propagating crack. Nat. Commun..

[38] Pan, X., Xiong, Q. & Wu, Z. New method for obtaining the homogeneity index m of Weibull distribution using peak and crack-damage strains. *Int. J. Geomech.*10.1061/(ASCE)GM.1943-5622.0001146 (2018).

[39] Xiong Q, Lin Q, Hampton JC (2021). Temporal evolution of a shear-type rock fracture process zone (FPZ) along continuous, sequential, and spontaneous well-separated laboratory instabilities-from intact rock to thick gouged fault. Geophys. J. Int..

[40] Xiong Q, Hampton JC (2021). A laboratory observation on the acoustic emission point cloud caused by hydraulic fracturing, and the post-pressure breakdown hydraulic fracturing re-activation due to nearby fault. Rock Mech. Rock Eng..

[41] Hampton J, Gutierrez M, Matzar L (2019). Microcrack damage observations near coalesced fractures using acoustic emission. Rock Mech. Rock Eng..

[42] Lin Q, Wan B, Wang S, Li S, Fakhimi A (2019). Visual detection of a cohesionless crack in rock under three-point bending. Eng. Fract. Mech..

[43] Lin Q, Wan B, Wang Y, Lu Y, Labuz JF (2019). Unifying acoustic emission and digital imaging observations of quasi-brittle fracture. Theor. Appl. Fract. Mech..

[44] Lin Q, Yuan H, Biolzi L, Labuz JF (2014). Opening and mixed mode fracture processes in a quasi-brittle material via digital imaging. Eng. Fract. Mech..

[45] Zhuang, J. & Touati, S. Stochastic simulation of earthquake catalogs. *Commun. Online Resourc. Stat. Seismicity Anal.*10.5078/corssa-43806322 (2015).

[46] Ross GJ (2021). Bayesian estimation of the ETAS model for earthquake occurrences. Bull. Seismol. Soc. Am..

[47] Mizrahi, L., Nandan, S. & Wiemer, S. Embracing data incompleteness for better earthquake forecasting. *J. Geophys. Res. Solid Earth*10.1029/2021jb022379 (2021).

[48] Baiesi M, Paczuski M (2004). Scale-free networks of earthquakes and aftershocks. Phys. Rev. E Stat. Nonlin. Soft Matter Phys..

[49] Zaliapin I, Gabrielov A, Keilis-Borok V, Wong H (2008). Clustering analysis of seismicity and aftershock identification. Phys. Rev. Lett..

[50] Davidsen J (2017). Triggering processes in rock fracture. Phys. Rev. Lett..

[51] Zaliapin I, Ben-Zion Y (2013). Earthquake clusters in southern California I: Identification and stability. J. Geophys. Res. Solid Earth.

[52] Zaliapin I, Ben-Zion Y (2013). Earthquake clusters in southern California II: Classification and relation to physical properties of the crust. J. Geophys. Res. Solid Earth.

[53] Keranen KM, Weingarten M, Abers GA, Bekins BA, Ge S (2014). Sharp increase in central Oklahoma seismicity since 2008 induced by massive wastewater injection. Science.

[54] Veen A, Schoenberg FP (2012). Estimation of space–time branching process models in seismology using an EM–type algorithm. J. Am. Stat. Assoc..

[55] Xiong, Q., Brudzinski, M. R., Gossett, D., Lin, Q., & Hampton, J. C. Seismic magnitude clustering is prevalent in field and laboratory catalogs [DATA] [Data set]. zenodo. 10.5281/zenodo.7328586 (2020). 10.1038/s41467-023-37782-5 PMC10097663 37045820

