# Supplementary material for: Seismic magnitude clustering is prevalent in field and laboratory catalogs
Source: Nat Commun. 2023 Apr 12;14:2056. doi: 10.1038/s41467-023-37782-5 (PMC10097663; doi:10.1038/s41467-023-37782-5)
Supplement: Supplementary file 1 — Supplementary Information [file 41467_2023_37782_MOESM1_ESM.docx]

# Supplementary Information

## Supplementary Note 1. Supplementary Figures and Text for the Investigations of Field Catalogs

Figure S1: Frequency-magnitude Distributions of Field Catalogs.


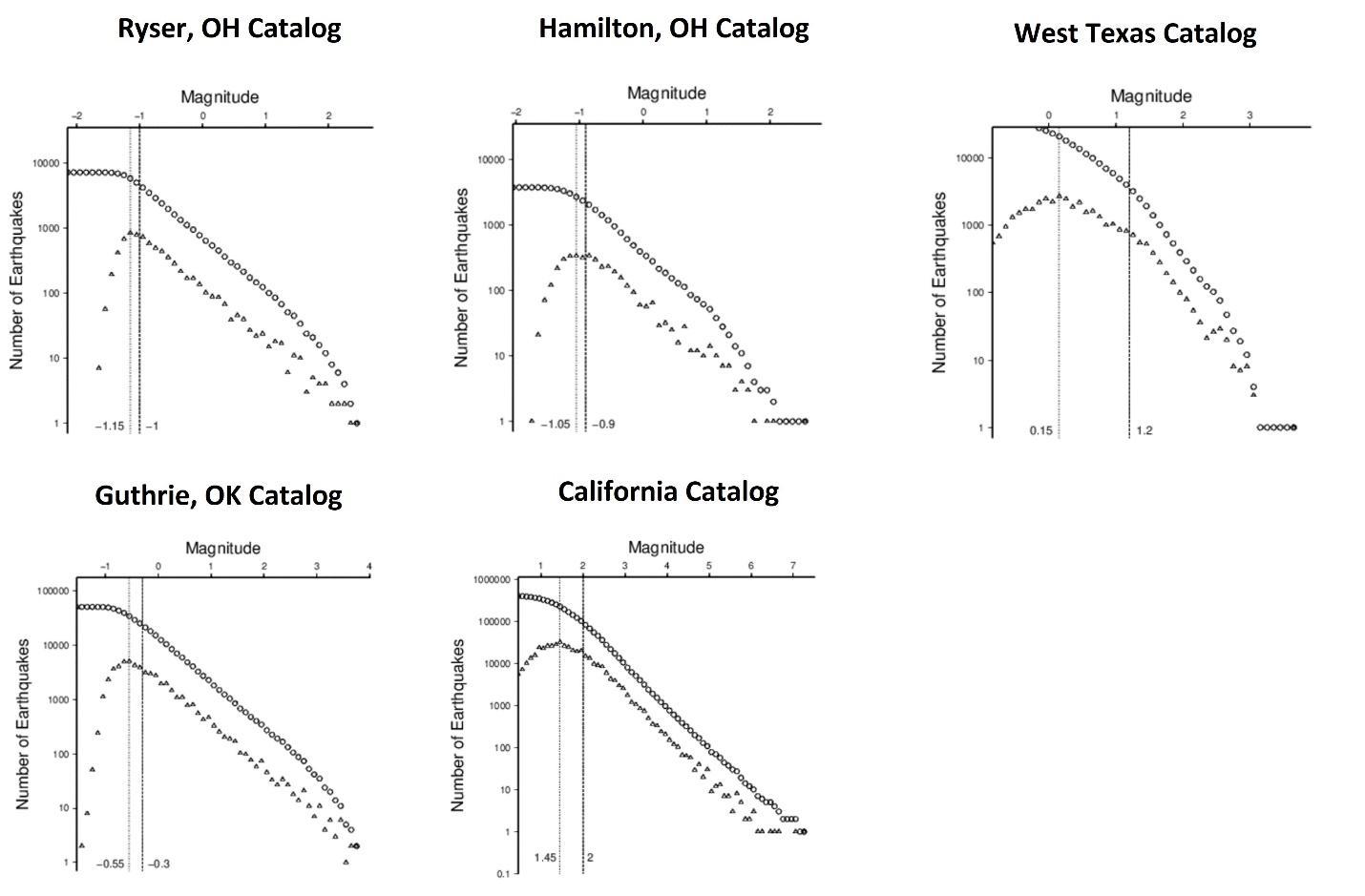


Fig. S1 Frequency-magnitude distribution (FMD) of each catalog, used to determine the catalog-specific magnitude of completeness ($m_{c}$). The lighter vertical dotted line depicts the FMD using the maximum curvature method. The darker vertical dotted line depicts the FMD using the b-value stability method. The latter method was used for our analysis in order to provide a more conservative estimate of the $m_{c}$. Regardless of the FMD method chosen, the magnitude clustering signature remains prominent even after applying our filtering methods.

Figure S2: Cumulative Distribution of California Catalog Subdivisions


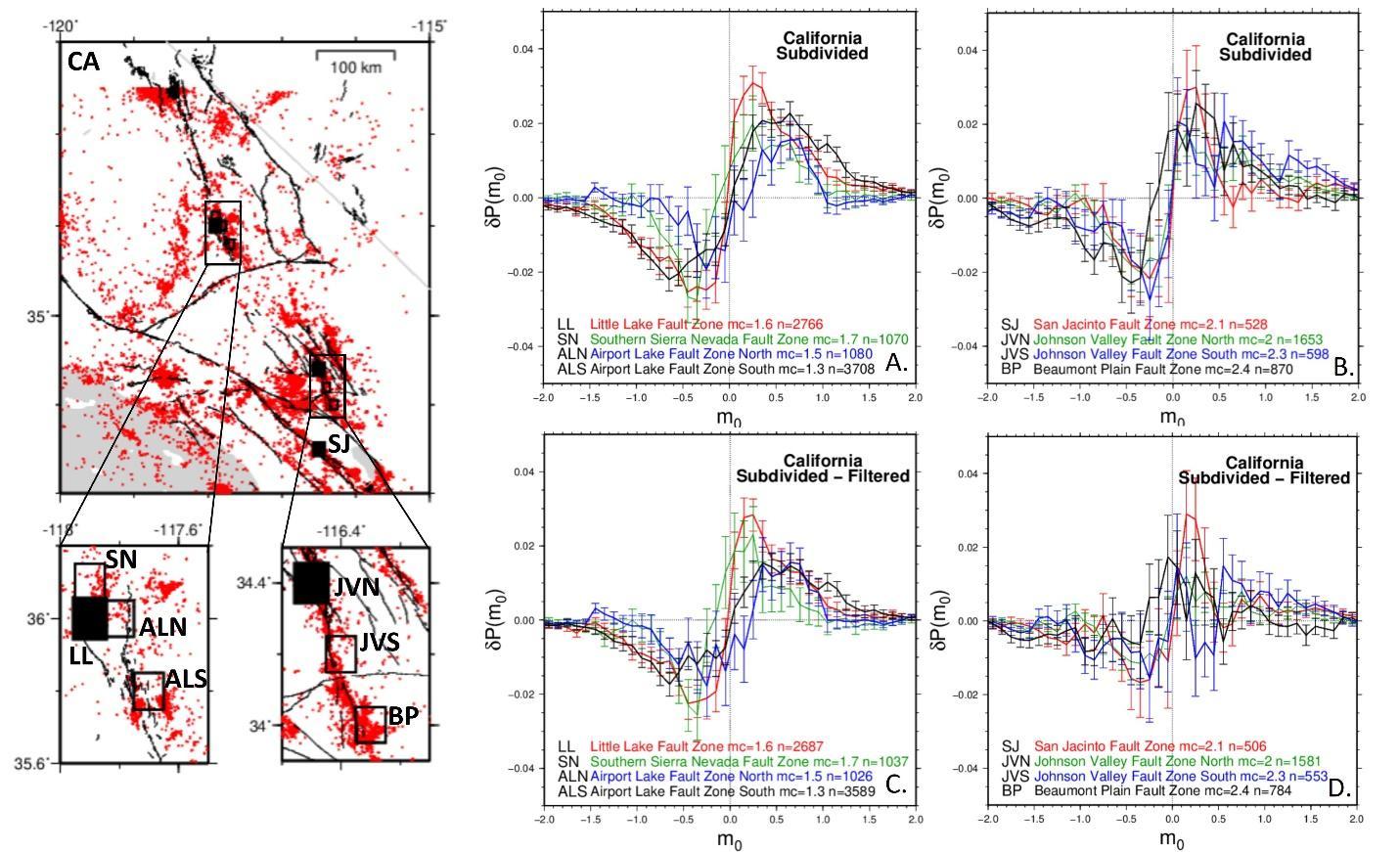


Fig. S2 Cumulative distribution for 8 areas of 10 sq. km in Southern California with pronounced seismicity. Figures A and B show 4 areas each before filtering is applied. Figures C and D show these same areas after applying magnitude of completeness and interevent time filters to correct for STAI. This analysis was done to more directly compare the California catalog to the induced catalogs in terms of spatial extent and number of events. $m_{c}$ = magnitude of completeness, n = number of events. The magnitude clustering signature in the tectonic California catalogs remains significant at these spatial scales. The maps are plotted using the Generic Mapping Tools software (<https://www.generic-mapping-tools.org/>), and the coastline database GSHHG (formerly GSHHS) is compiled from the World Vector Shorelines.  See The Global Self-consistent, Hierarchical, High-resolution Geography Database (GSHHG) for further details (<https://docs.generic-mapping-tools.org/6.2/datasets/gshhg.html>).  The US state boundaries are from the National Oceanic and Atmospheric Administration database (<https://www.nohrsc.noaa.gov/data/vector/master/st_us.kmz>).

Figure S3: Cumulative Distribution of Field Catalogs


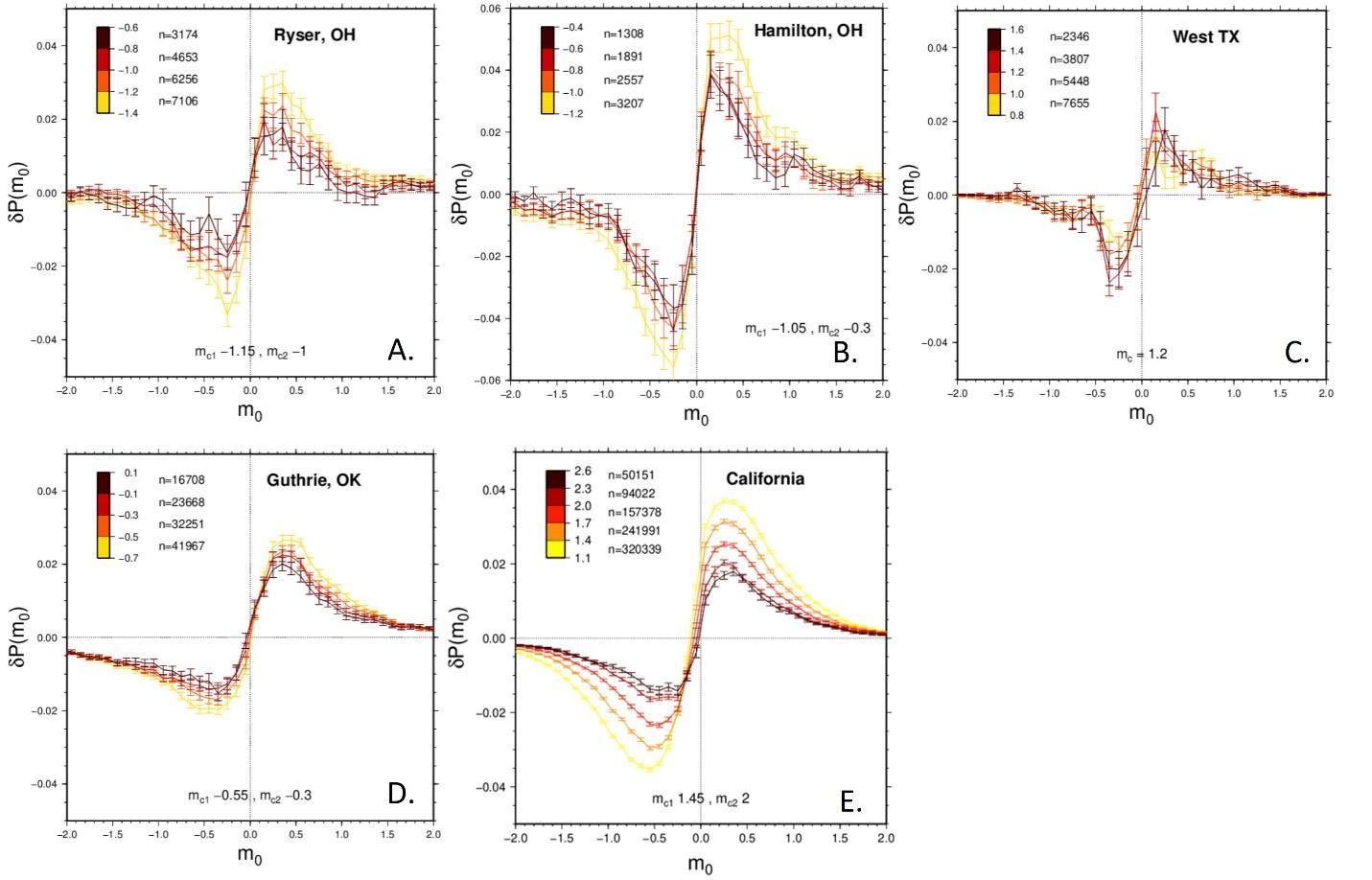


Fig. S3 Cumulative distribution of difference in probability between the observed catalog and a randomized version, $\delta P\left( m_{0} \right)$, as a function of magnitude difference ($m_{0}$), for each catalog A-E before any filtering is applied. Magnitude threshold ranges were chosen based on the magnitude-frequency distribution for each specific catalog. $m_{c}=$ magnitude of completeness, n = number of events. In the case of catalogs with two magnitude of completeness values, the larger value was chosen for our analysis.

Figure S4: Cumulative Distributions of Field Catalogs after Applying Filters


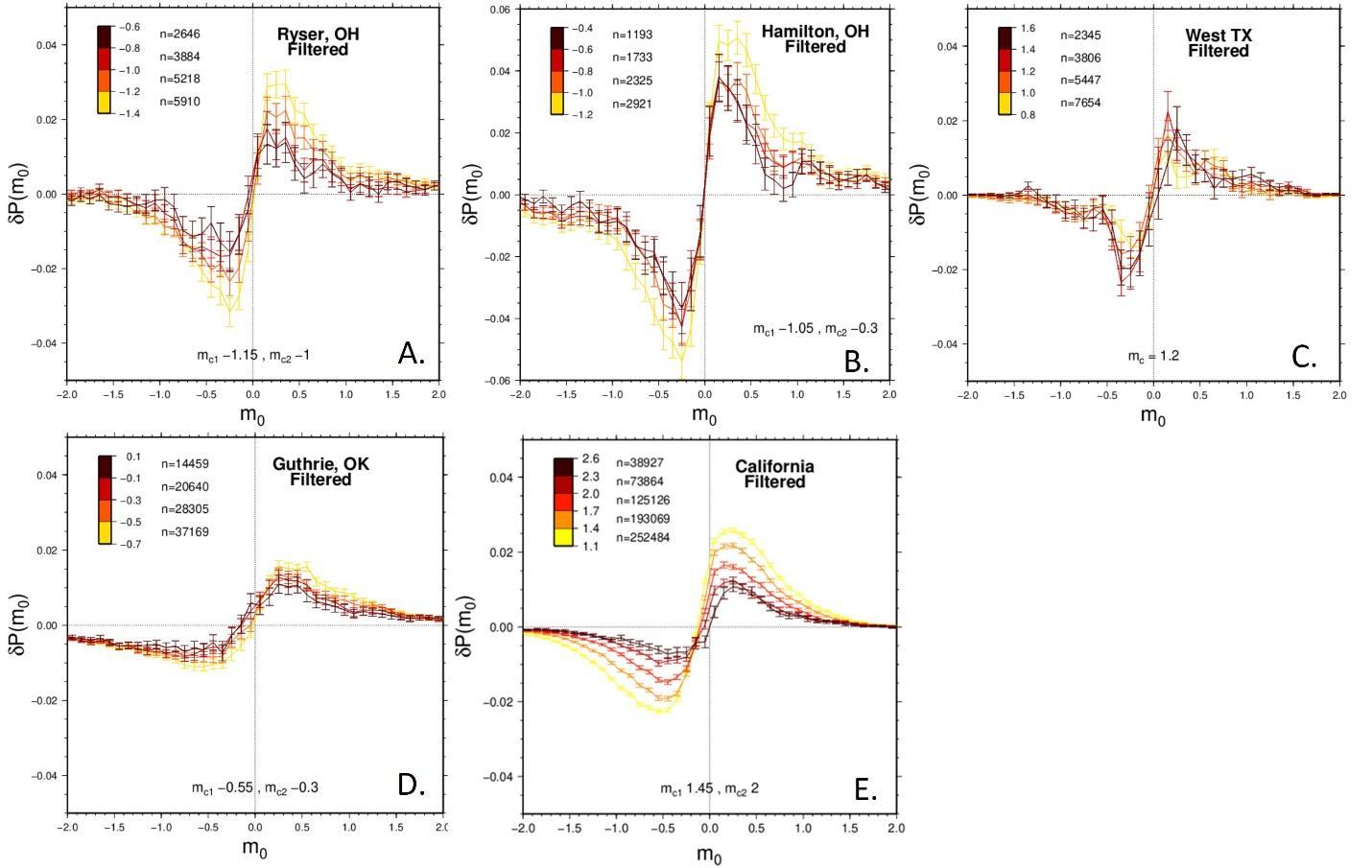


Fig. S4 Cumulative distribution for each catalog A-E after applying magnitude of completeness and interevent time filters to correct for STAI. Same conventions as figure S2. Interevent time filters were set for each specific catalog based on how quickly events repeat in that catalog, found by waveform analysis. We used a 10-second filter for the Guthrie catalog (D), as this catalog was the most quickly repeating. We used a 30-second filter for the West Texas (C), Ryser (A), and Hamilton (B) catalogs. We used a 2-minute filter for the California (E) catalog. The number of events for the filtered West Texas (C) catalog only decreases by one event compared to the unfiltered. This is due to the template matching method being used for this catalog not detecting events separated by less than 30 seconds. The magnitude clustering signature decreases to varying degrees based on the catalog after this filtering is applied, but in all cases remains significant.

Figure S5: Non-cumulative Distribution of Field Catalogs


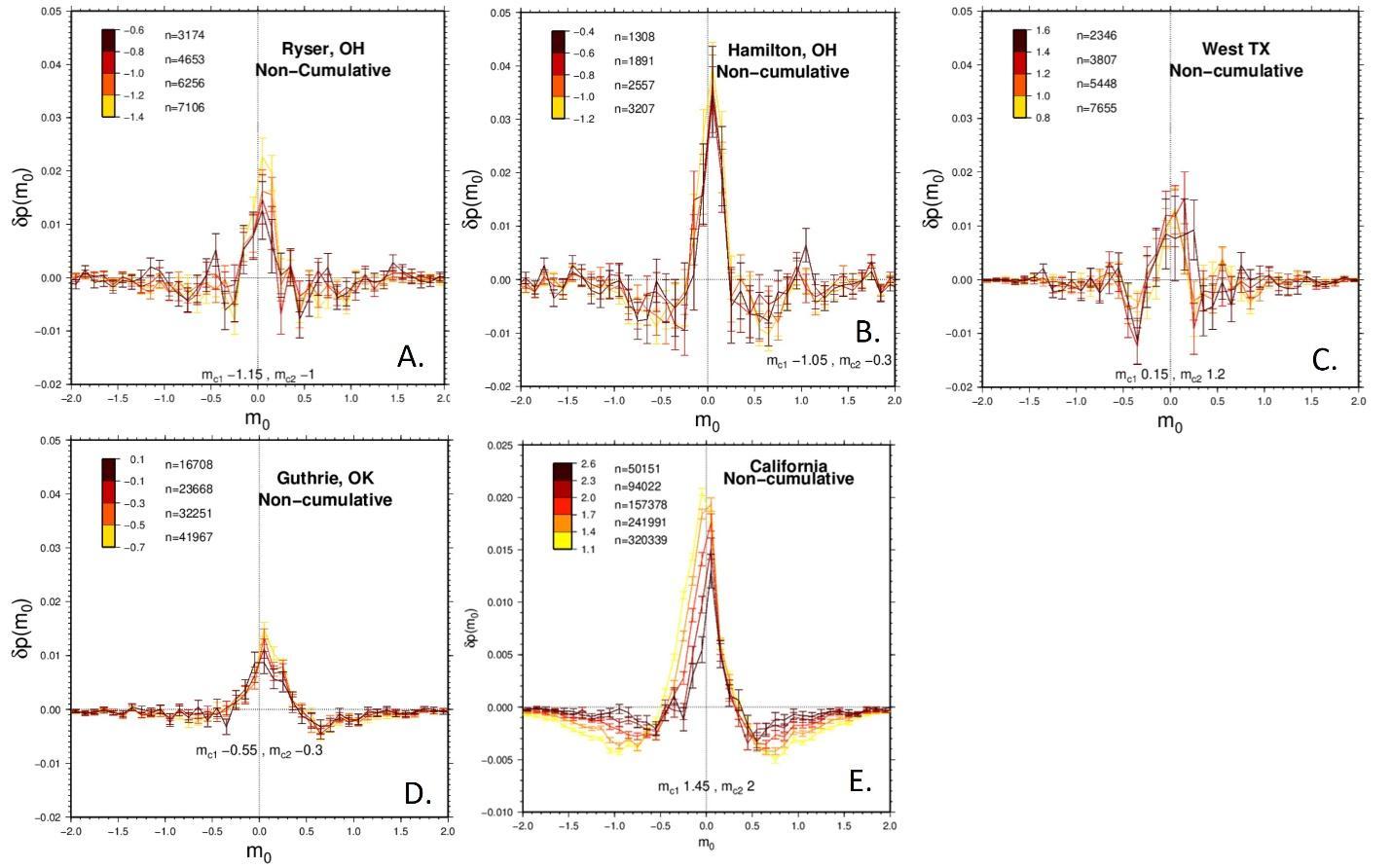


Fig. S5 Non-cumulative distribution for each catalog A-E before any filtering is applied. Same conventions as the cumulative distribution figures. For the non-cumulative case, the real catalog is compared with the randomized versions of the catalog at each magnitude difference bin.

Figure S6: Non-cumulative Distribution of Field Catalogs after Applying Filters


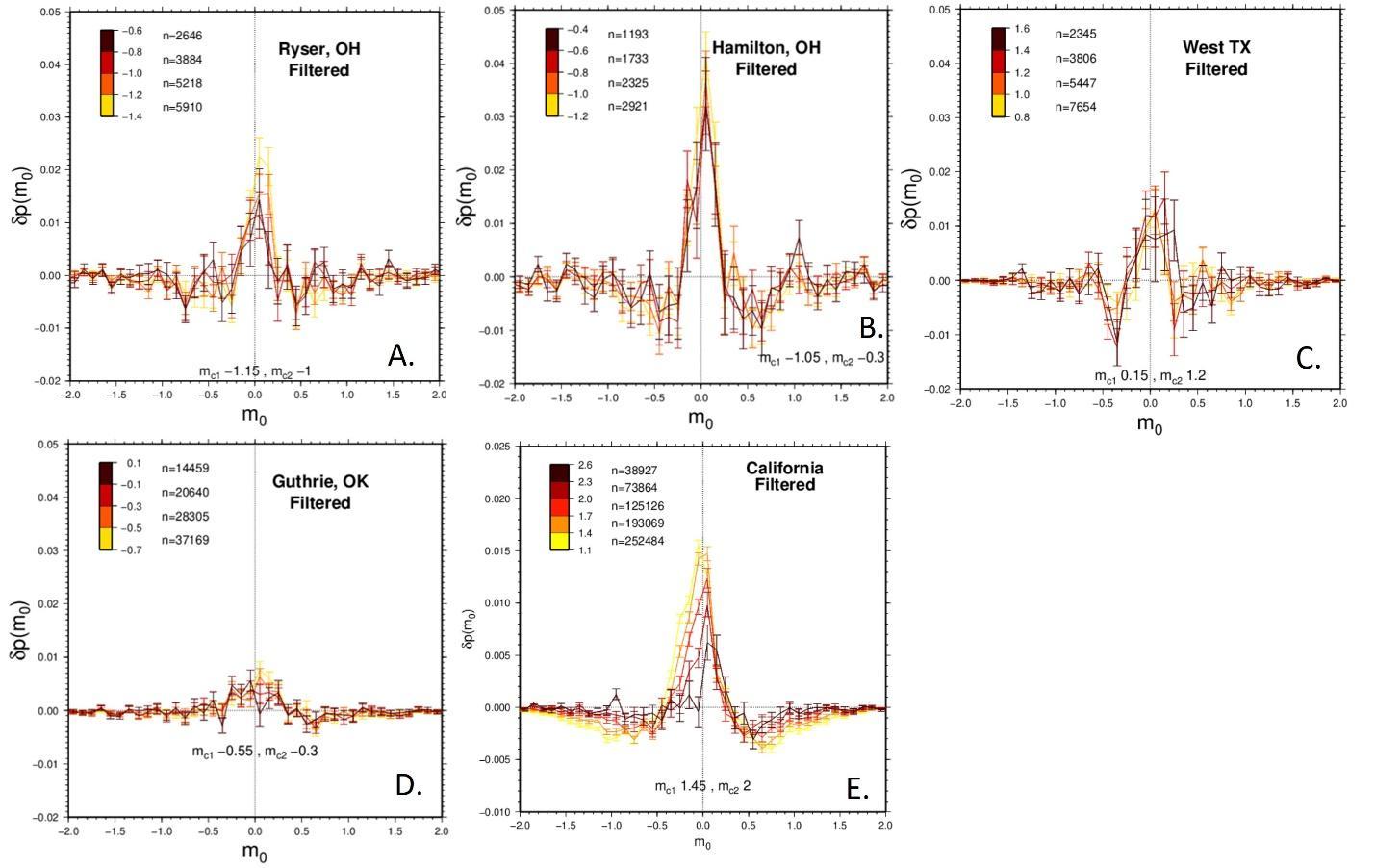


Fig. S6 Non-cumulative distribution for each catalog A-E after applying magnitude of completeness and interevent time filters to correct for STAI.

Figure S7: Comparison of Field Catalog Non-cumulative Distribution


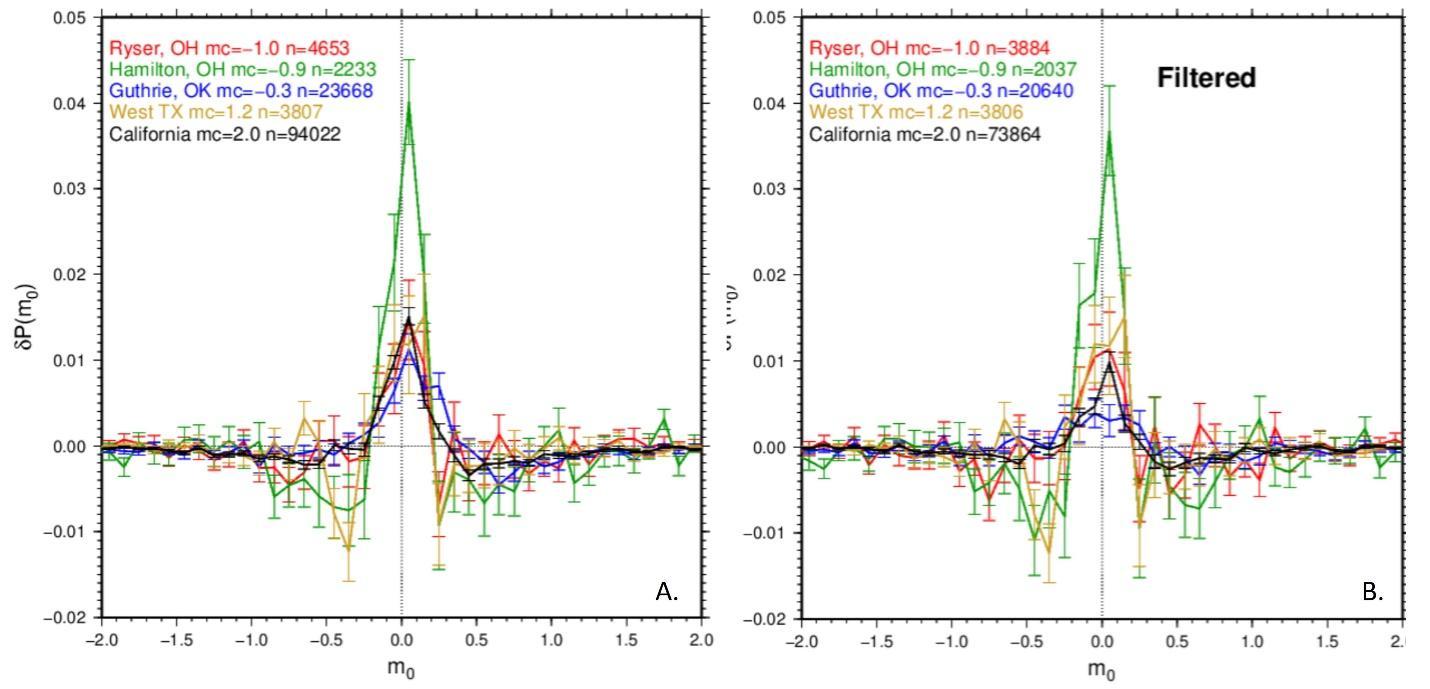


Fig. S7 Comparison of the non-cumulative distribution for each catalog (Ryser, Hamilton, Guthrie, West Texas, and California) before filtering (A) and after (B). The magnitude clustering signature decreases after filtering but remains significant for all cases.

Figure S8: Cumulative Distribution of Field Catalogs with 3 Standard Deviation Confidence Interval


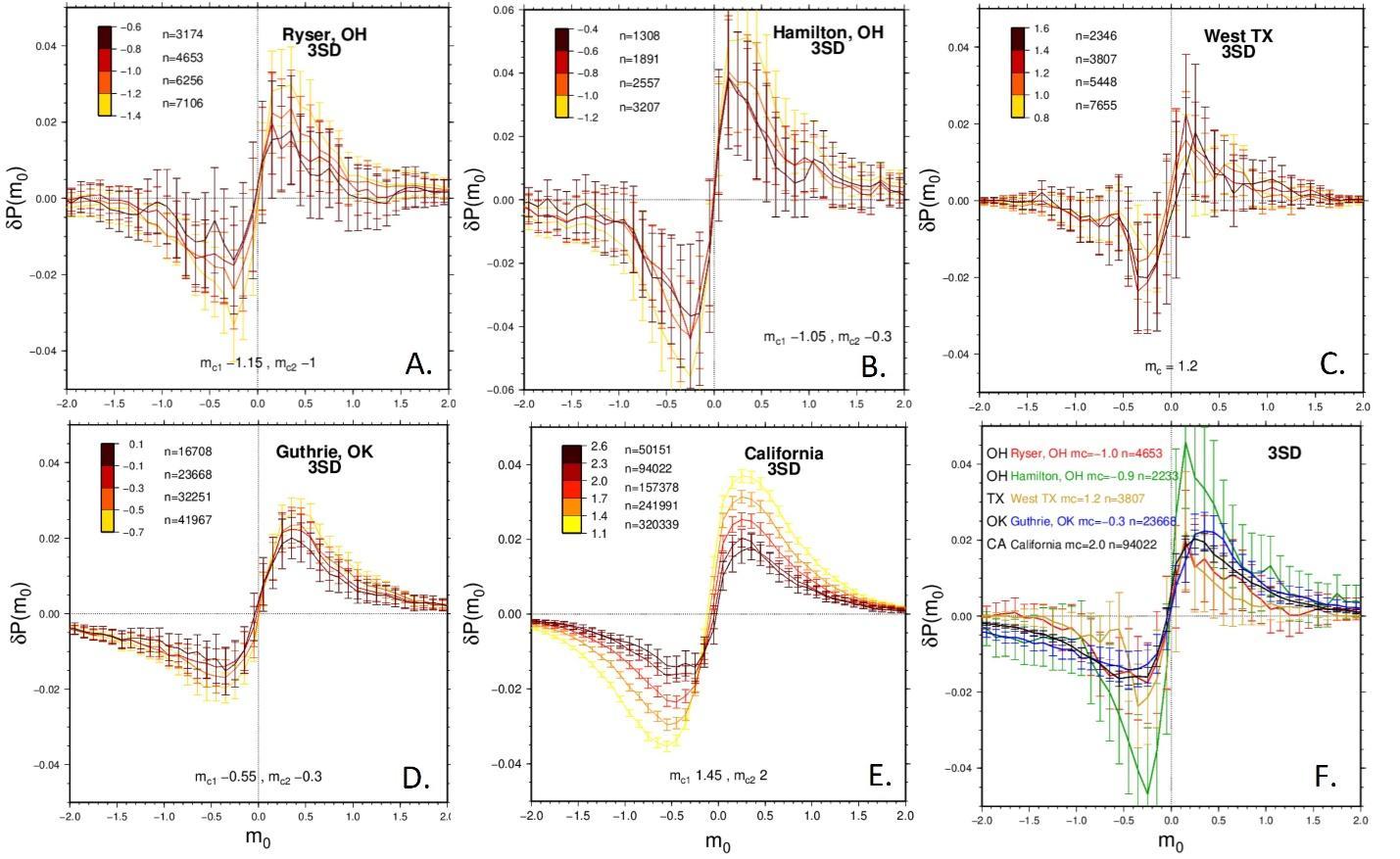


Fig. S8 Cumulative distribution using a 3 standard deviation confidence interval (compared to 1 standard deviation for previous figures in main text and supplementary) for each catalog (A-E), and a comparison between catalogs (F) before applying any filters. Same conventions as Fig. S3.

Figure S9: Cumulative Distribution of Field Catalogs with 3 Standard Deviation Confidence Intervals after Applying Filters


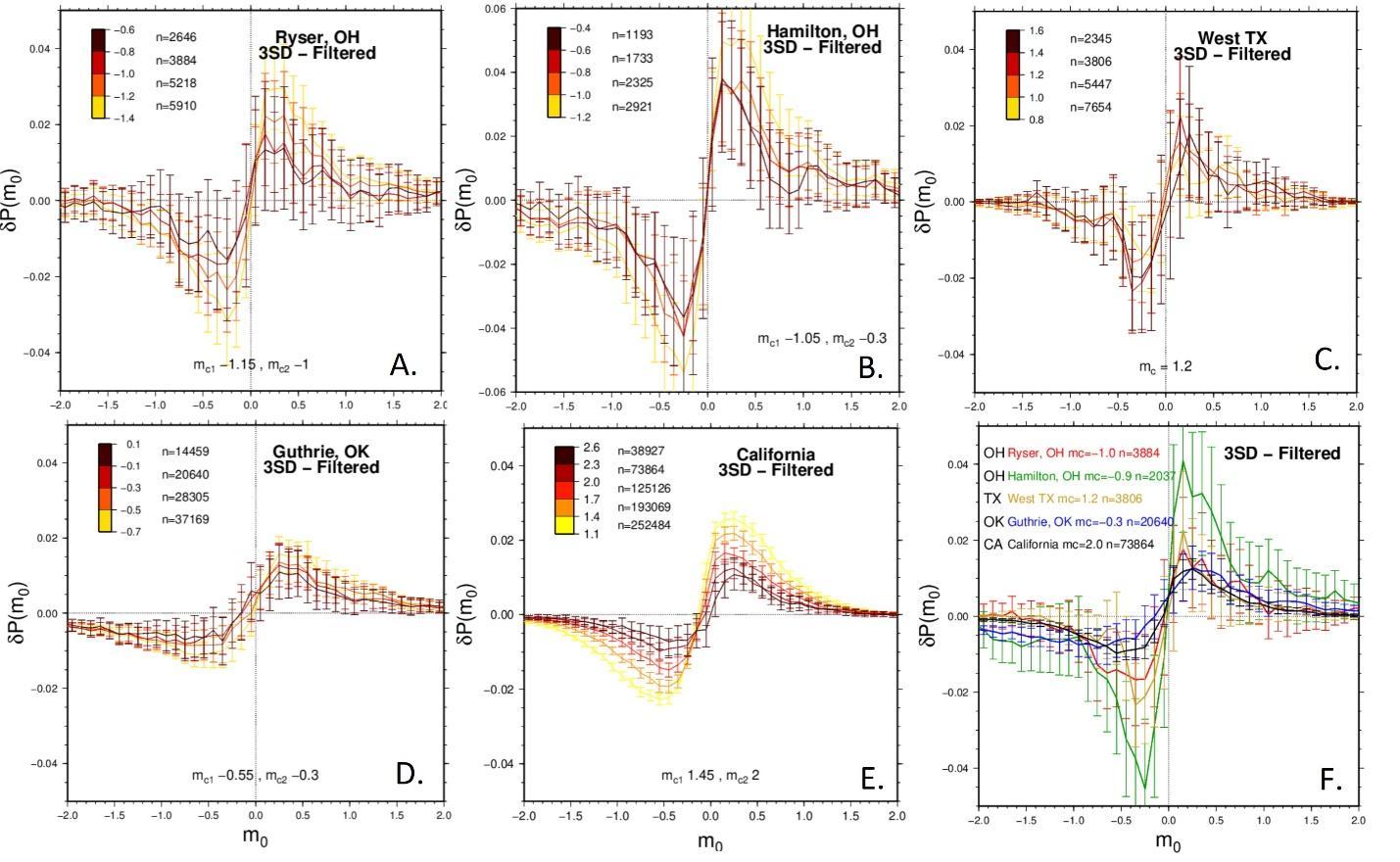


Fig. S9 Cumulative distribution using a 3 standard deviation confidence interval for each catalog (A-E), and a comparison between catalogs (F) after applying magnitude of completeness and interevent time filters to correct for STAI. The magnitude clustering signature remains significant using this higher standard deviation for all catalogs with the exception of the Ryser, OH catalog. This is most likely due to the small number of events combined with the Ryser catalog already displaying a lower clustering signature than the Hamilton, OH catalog.

Figure S10: ECDF Plots of Field, Laboratory, and Synthetic Catalogs


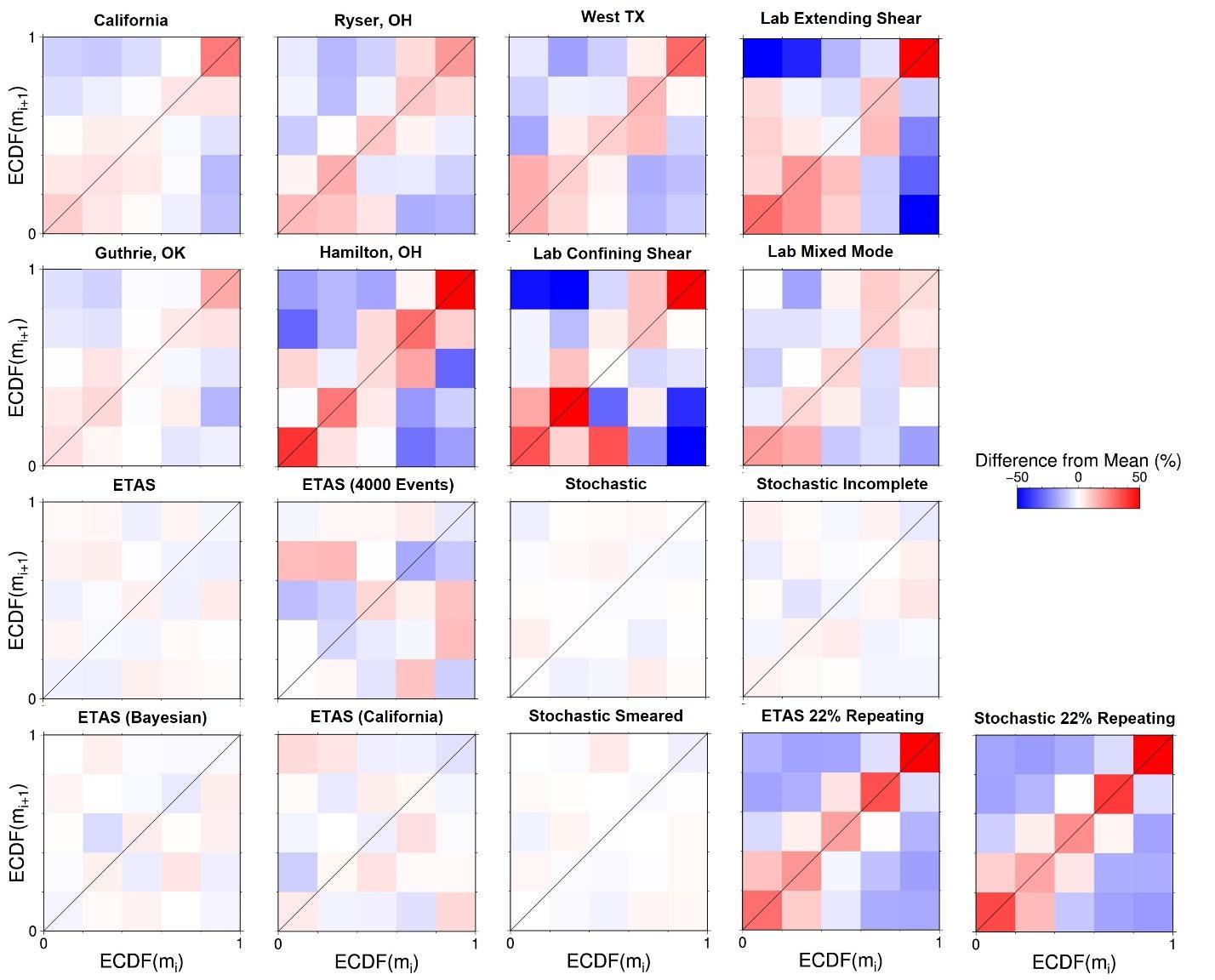


Fig. S10. We have also developed a new strategy to illustrate the non-random relationship between subsequent event magnitudes in our field and laboratory catalogs using the empirical cumulative density function (ECDF) for the magnitude distribution. The ECDF is calculated by sorting the catalog magnitudes from smallest to largest and assigning a value equal to the count divided by the total number. The catalog is then resorted by time and the ECDF value of each event (i) is compared to the ECDF value of the subsequent event (i+1). The results are divided into bins of 0.2 x 0.2, calculating the number of events that fall into each bin. To establish what are potential variations from the magnitude distribution alone, ECDF values are also calculated on the catalog randomized by time, and we then measure the mean number across the bins of the randomized version. Colors show the percentage difference of the value in each bin relative to the mean established from the randomized catalog. For field and laboratory catalogs, subsequent events with the same ECDF bin value (diagonal line) occurred at higher rates than randomized catalogs, exceeding 50% in some cases. In comparison, seven different strategies for constructing synthetic catalogs were unable to produce this pattern (ETAS by Mizrahi et al.^1^ for 30,000 and 4,000 events; Zhuang and Touati^2^ for 100,000 events; Zhuang and Touati^2^ with 25,000 events with artificial incompleteness; Zhuang and Touati^2^ for 100,000 events with magnitudes smeared with σ of 0.2 to represent uncertainty; Bayesian ETAS by Ross^2^ for 20,000 events; Bayesian ETAS by Ross^2^ using parameters tuned to the California catalog for 12,000 events). However, we also constructed a pair of catalogs where we artificially inserted a repeated event magnitude (with magnitude smearing) for 22% of the events in both the ETAS and stochastic catalogs resembles the pattern observed for the Hamilton catalog.

Figure S11: Time-range Magnitude Clustering Analysis Using Both Cumulative Distribution and ECDF Plots


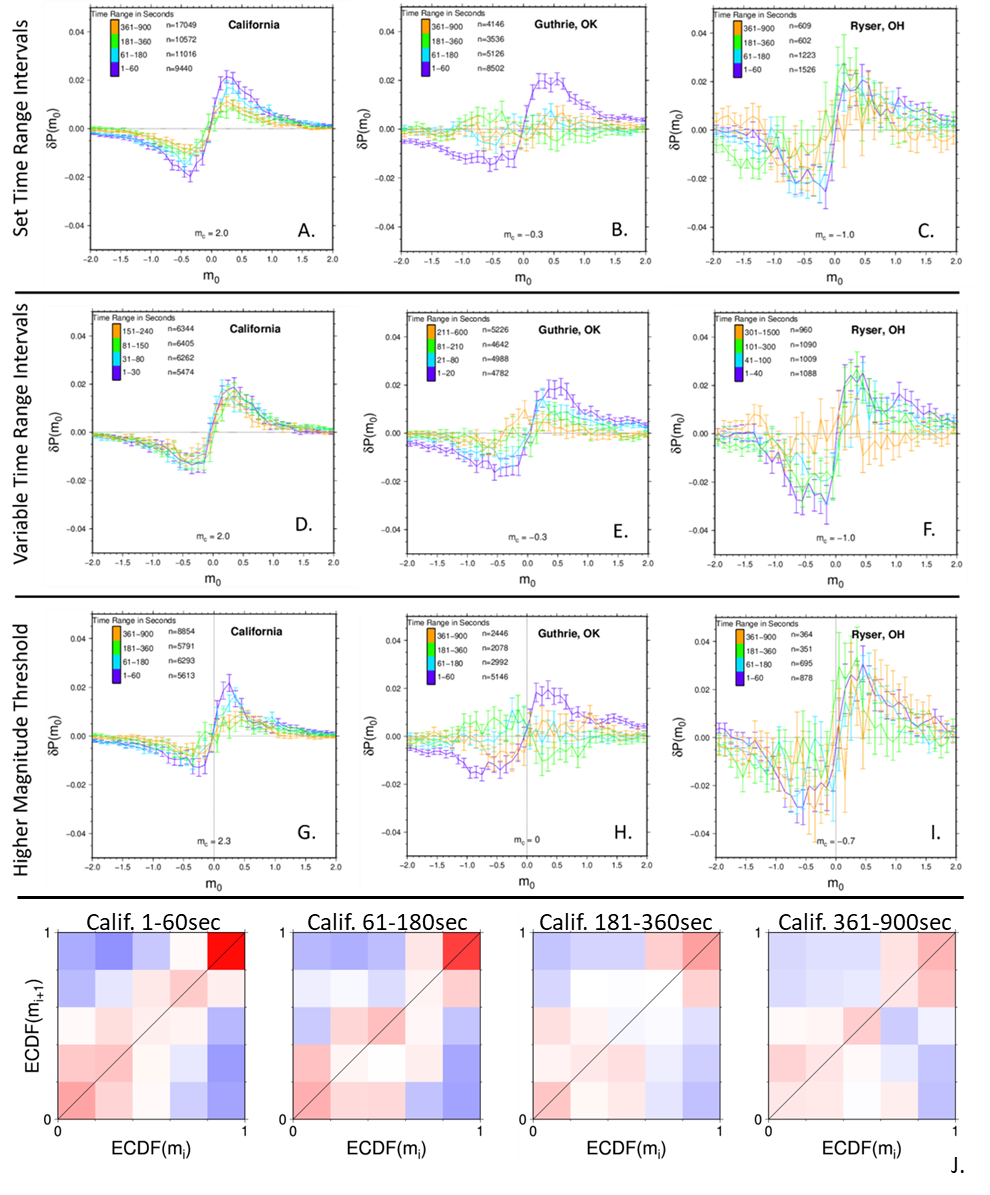


Fig. S11 Comparison of two different methods for separating analysis into time ranges. n = number of events, $m_{c}=$ magnitude of completeness. A-C used set time range intervals for each catalog, with a more widely varying value of n. D-F focus on maintaining a similar n value for each time range within a catalog, therefore the time ranges vary between catalogs. The limited number of events for the Ryser catalog using both methods causes the large standard deviation demonstrated using the set time range method, so this method was chosen for our analysis in the main text. Figures G-I show the same analysis as A-C but with a higher magnitude threshold applied. Figure J shows ECDF plots of the STAI filtered California catalog at each of the time range intervals.

## Supplementary Note 2. Supplementary Figures and Text for the Investigations of Laboratory Catalogs

### II-1. Data Analysis

The statistical significant magnitude clustering or non-clustering is detected by the features of cumulative probabilities of observing a magnitude difference (M_0_) between consecutive energy releases of laboratory catalogs. For a data set of energy releases, AE for laboratory-scale or seismic catalog for field-scale, a magnitude difference between the *i*^th^ and the (*i*+1)^th^ energy releases is defined as: ΔM*_i_* = M*_i_*_+1_ – M*_i_*, where M*_i_*_+1_ – M*_i_* are the magnitudes of the two energy releases, respectively. M_0_ is the scale for ΔM. The cumulative probability of observing a magnitude difference on M_0_ scale defines as, *P*(ΔM < M_0_). For the randomized data set M*^*^*, we write ΔM*_i_^*^* = M*_i_^*^* – M*_i_*. The difference of the cumulative probabilities between the real and randomized data sets (i.e. *δP* = *P*(ΔM < M_0_) – *P^*^*(ΔM*^*^* < M_0_) should be of no statistical significance once non-clustering exists, and should be of clear statistical significance once clustering exists. After large number of randomizations, i.e., 1,000 or 10,000 times, the cumulative probability differences (*δP*) as well as their standard deviations on each M_0_ intervals can be evaluated, so as the condition of non-clustering or significant clustering for the data set.

### II-2. Clustering and Non-clustering Definitions

Presently we define the clustering of statistically significance as exceeding 3-standard deviations. However, using 2- or 3-standard deviations is of only secondary influence on the rock fractures not under shear stress dominated environment. The tendency of increasing significance can clearly be observed once we narrow the investigated inter-event distance close to the characteristic length of geometric constraints. This increase is so prominent at the moment the inter-event distance approaches such characteristic length that defining “significant” as 2- or 3-standard deviations only slightly alter the first observation of significant clustering.

### II-3. Data Acquisition Systems

Noting that the laboratory tests are accumulated across a decade, due to the differences of data acquisition systems and acquisition settings, the estimations on the AE magnitude must be different. For the data set provided by refs^3,4^, in-situ calibration has been conducted and the AE magnitudes can be estimated most accurately on the first peaks of the signals. For other tests, this estimation must be based on the overall peak amplitude of the signals. We validate both estimations are effective for detecting significant clustering or non-clustering, by removing the calibration coefficients obtained for refs^3,4^ and re-analyzing using overall peak amplitude of the signals. The factors for determining the significant clustering or non-clustering can be de-coupled based on the observations across different data acquisition systems and settings providing further convincing evidence for the de-coupling process.

### II-4. Constrained Interevent Correlation

Investigation on the conditioned inter-event distance follows Ref.^5^. It investigates the probability differences (*δP*) on a refined data set, which writes:

*δP*(M_0_|r_0_) = *P*(ΔM < M_0_|Δr < r_0_) – *P^*^*(ΔM*^*^* < M_0_|Δr < r_0_) (1)

where Δr represents the distance between the events, i.e., Δr_i_ = r_i+1_ – r_i_, and for the randomized data set, again, Δr_i_*^*^* = r_i_*^*^* – r_i_.

In our investigation, we progressively change the parameter r_0_. In Ref.^5^, this conditioning treatment is devised for investigating the influence of the Short-Term Aftershock Incompleteness (STAI). However, within laboratory settings where the constraint conditions are well-defined, the significant magnitude clustering always occurs once the parameter r_0_ approaches the characteristic length of the geometric constraint regardless the types of constraints, e.g., pre-cut fault or the specimen size related constraint.

For instance, the characterization lengths of tensile stressed rock fractures are different among different tests. It can be ~ 30 mm for 4-point bending tests conducted at Colorado School of mines or 3-point bending tests conducted at University of Minnesota^6,7^, and ~ 90 mm for hydraulic fracturing tests (Fig. S12b) conducted at Halliburton^8,9^, and much smaller for the tensile-wing fractures whose test was conducted at Nanyang Technological University, Singapore, and the specimen thickness was smaller^10^.

### II-5. Constrains for Confined and Extending Shear Rock Fractures

The energy releases from the confined shear rock fracture^4^ are from a rectangular area of 25 mm length and 30 mm thickness. The specimen thickness is larger than the confined length, therefore does not act as a geometric constraint. The constraint is also not a factor that induce magnitude clustering for the extending shear rock fracture^3^. It is because we can again observe significant magnitude clustering within sectional investigations (i.e. an squared patch of rock fracture) of the energy releases.

### II-6. Conditional Clustering Observations

We first observe the unconditional magnitude clustering in shear stressed rock fractures. Whether the shear rock fractures are strictly confined or extending will not affect the result. We observe, regardless of whether we exclude or not the events under the magnitude of completeness (Fig. S11a) and condition the time range of the randomized data set locally (illustrated in “Temporal Stability” of this supplementary material), Significant magnitude clustering can always be observed. Significant magnitude clustering can also always be observed for investigating the regional energy releases only, i.e., a square patch of the rock fracture (illustrated in “Regional Observation on Extending Rock Fracture under Shear Stress” of this supplementary material) or dropping the early-stage energy releases from the observation (also illustrated in “Regional Observation on Extending Rock Fracture under Shear Stress” of this supplementary material).


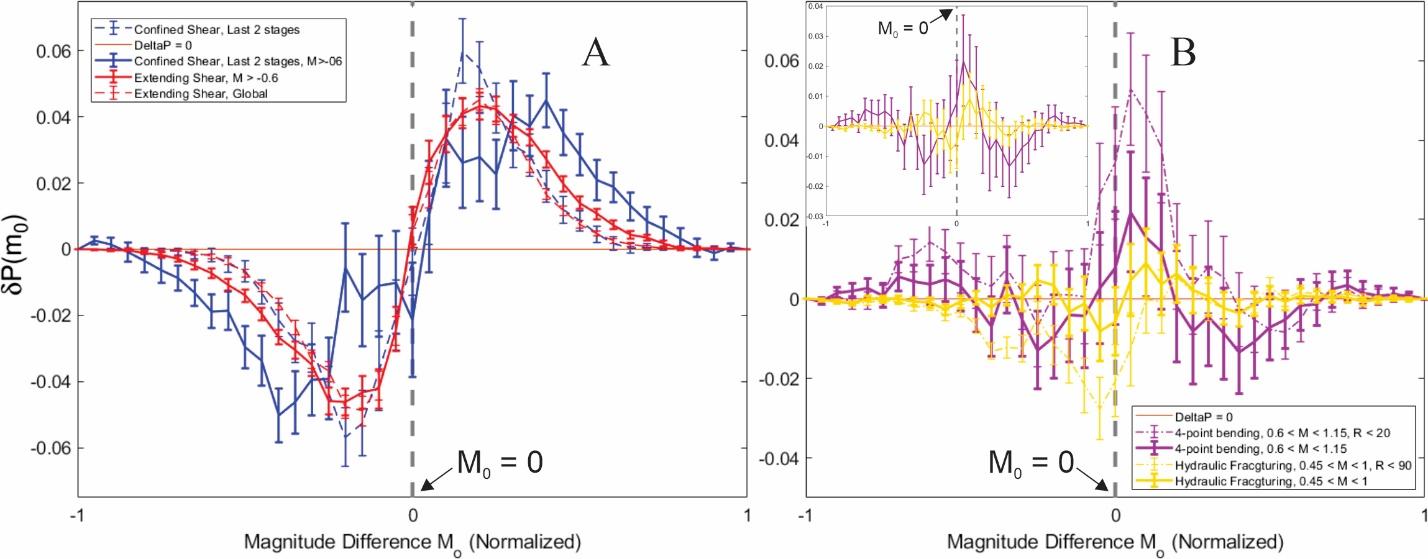


Fig. S12. A) significant magnitude clustering for extending and confined shear stressed rock fractures. B) non-clustering for 4-point bending and hydraulic fracturing energy releases (insert), and the significant clustering after conditioning the inter-event distances to the characteristic lengths of the geometric constraints, ~ 30 mm for 4-point bending, and ~ 90 mm for hydraulic fracturing tests. Error bars of 1 standard deviation are used. Examples within Fig. S11 are based on the energy releases within the linear section of the semi-log scale frequency-magnitude distributions, i.e. taking only events above the magnitude of completeness into the analysis. However, the conclusion on the conditions for distinguishing non-clustering or significant clustering based on all sources, or based on all sources above the magnitude of completeness are the same. The magnitudes of completeness for different data sets are relative values. Due to different rock types and data acquisition settings, test-specific magnitude of completeness are different.

The rock fractures not under shear stress conditions do, however, always display non-clustering globally (Fig. S11b). This observation is again, not affected by the events below magnitude of completeness; and again, temporally stable. The only, and always effective change that will make the magnitude clustering occur is to condition the inter-event distance (parameter r_0_) to the level approaching the characteristic length of the geometric constraints, regardless of whether the type of constraint consists of the pre-cut fault or the specimen’s smallest dimension, i.e., the thickness dimension. For all magnitude ranges, significant clustering will occur once the parameter r_0_ approaches the characteristic length of the constraints (for instance, see Fig. S12).


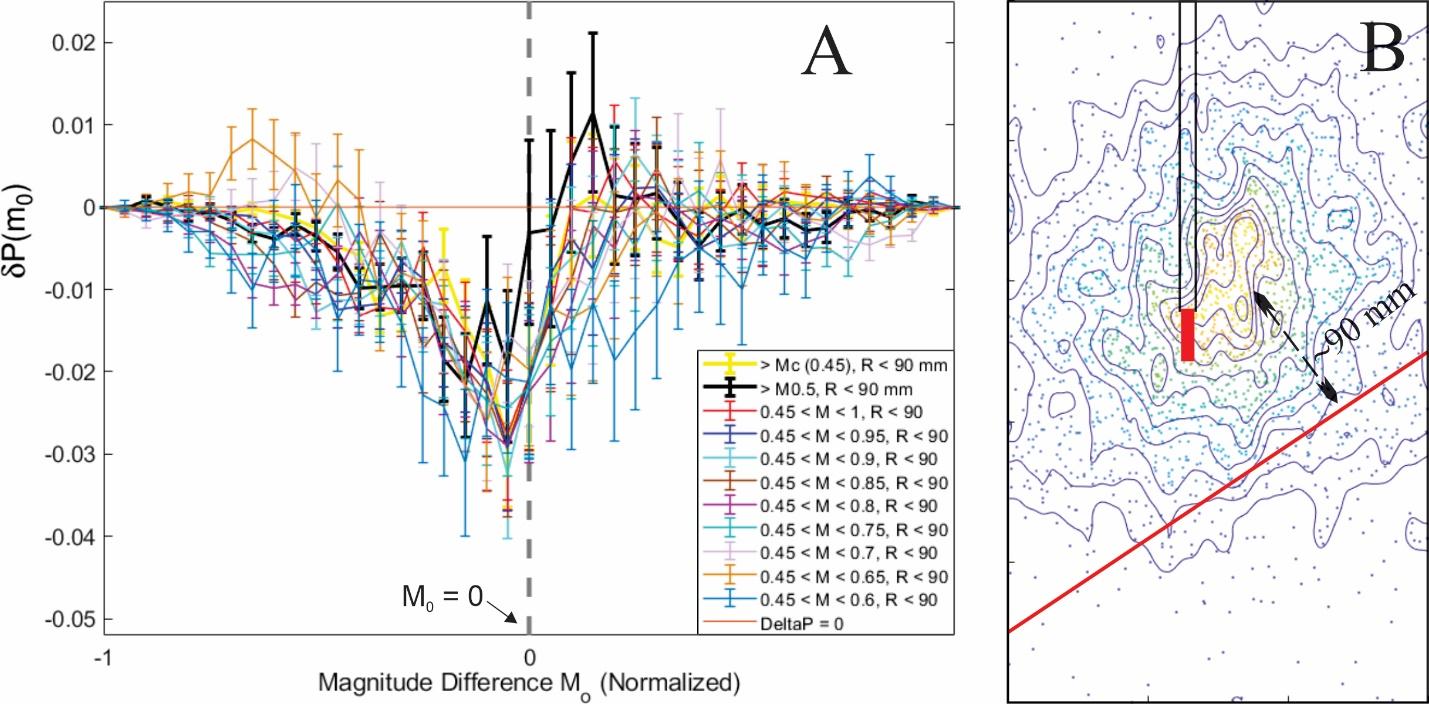


Fig. S13 A) significant clustering detected as investigated inter-event distance approaches 90 mm. Again, all display probability increases at M_0_ ~ 0. Error bars of 1 standard deviation are used. B) approximate distance between the point cloud of induced energy releases by hydraulic fracturing and the pre-cut fault (red line).

### II-7. Regional Observation on Extending Rock Fracture under Shear Stress

Here, we take only regional energy releases of the extending shear stressed rock fracture into investigation. For this testing configuration, the extending rock fracture encounters no constraints on its propagation direction. The only constraints are the specimen boundary and specimen thickness. Selecting a region that is at some distance away from the specimen upper boundary, significant clustering can be observed again (Fig. S13a). This observation rejects the hypothesis that it is only the geometric boundaries (i.e., considering the testing configuration for the confined shear stressed rock fracture) that impose the significant clustering observation. In contrast, non-clustering is the observation for tensile stress (3- or 4-point bending) and hydraulic fracturing (also tensile dominated) cases. As such, magnitude clustering appears to be unconditional for rock fractures under a shear stress condition. Further conditioning the inter-event distance for the shear stressed rock fractures can have secondary enhancement on the significance of magnitude clustering (Fig. S13b) once the conditioned inter-event distance approaches the smallest geometric constraint such as the specimen thickness. Testing configurations for the example tests will be illustrated in “Loading Curves, Energy Inputs for the Rock Fractures under Shear and Tensile Stress” of this supplementary material.


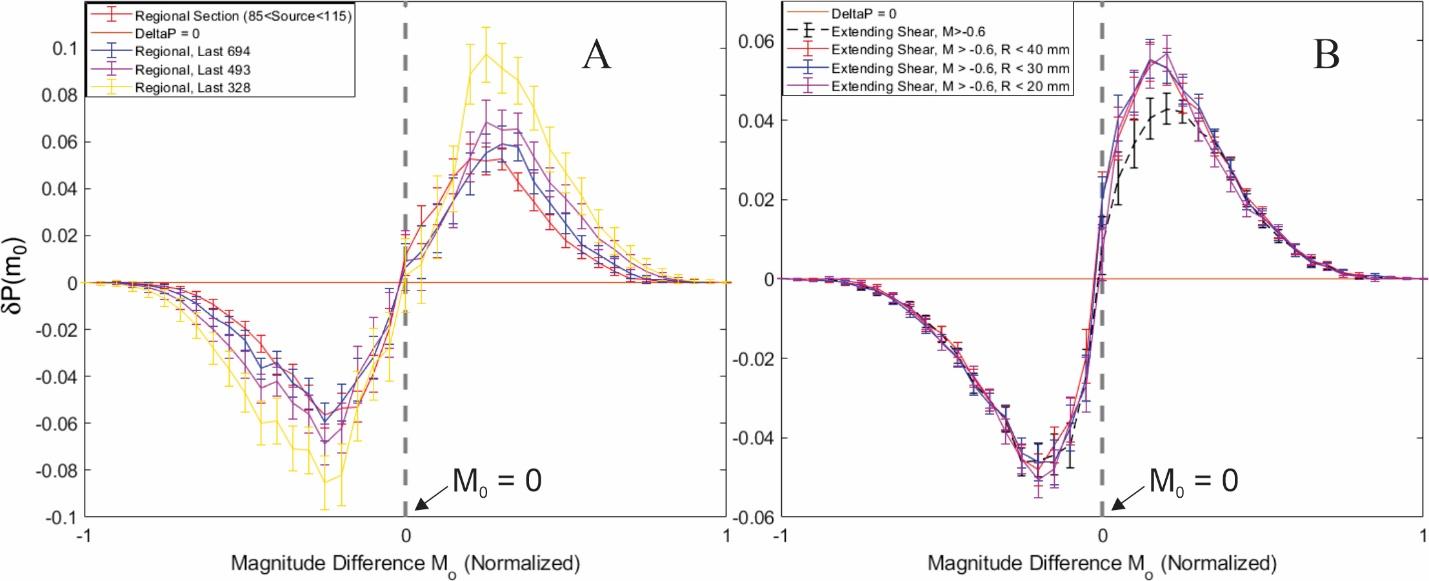


Fig. S14. A) Regional significant clustering for an extending shear stressed rock fracture. B) secondary enhancement on the significance of magnitude clustering once the conditioned inter-event distance approaches the specimen thickness. Error bars of 1 standard deviation are used.

### II-8. Temporal Stability

Investigation of temporal stable/unstable feature follows the method in Ref. ^11^. This method applies a window for the randomized magnitude (M*^*^*), selecting only the energy releases within the window specifying range for the randomization process. For instance, a window of 40 events will select M_i_*^*^* from only the 81-event set where its 41^st^ event is event *i*. The testing time or temporal density of energy releases will be affected by the loading protocols. Thus, for investigating the temporal stability, this approach has been used, instead of using an absolute time window of seconds for the investigations on field-scale catalogs. We confirm that non-clustering or significant clustering will not be altered by applying this event-window (Fig. S14a), unless this window contains too few events so that the range of 3 standard deviation covers everything.


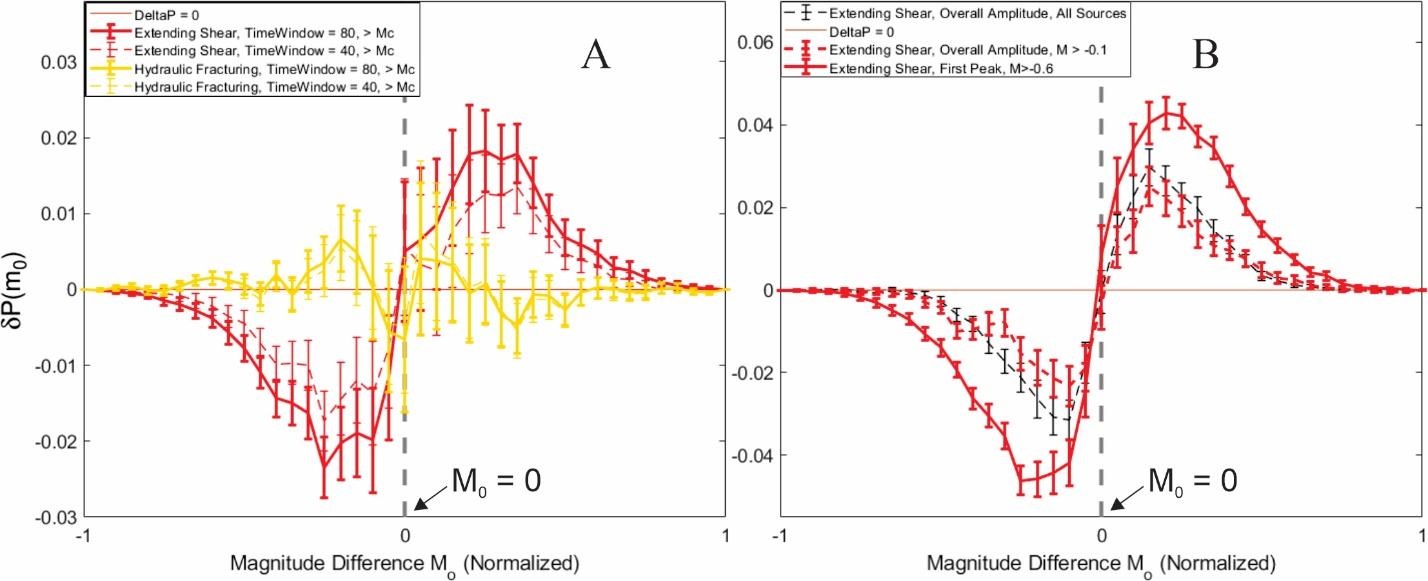


Fig. S15. A) Temporal stability feature of magnitude clustering/non-clustering. Given an extending shear fracture and a hydraulic fracture as the examples. However, this feature holds true for all other experimental cases investigated within this paper. B) observations using different methods to estimate the magnitudes. Given an extending shear fracture as the example. Error bars of 1 standard deviation are used.

### II-9. Loading Curve, Energy Inputs for the Rock Fractures under Shear and Tensile Stress

#### Extending shear:

Extending shear rock fractures were from Ref.^3^, and the unpublished ones with similar loading and geometry settings. The specimens were approximately 150 mm × 76 mm × 30 mm, with an inclined central open flaw (Fig. 4b). Specimens were uniaxially loaded along the longitudinal direction (i.e., the 150 mm axis). Most energy releases are within the post stress-peak loading domain (Fig. S15a). As such, the energy input is progressively decreasing during the energy release process. To exclude or not to exclude the energy releases before the stress-peak will not alter the observations of significant clustering or non-clustering.


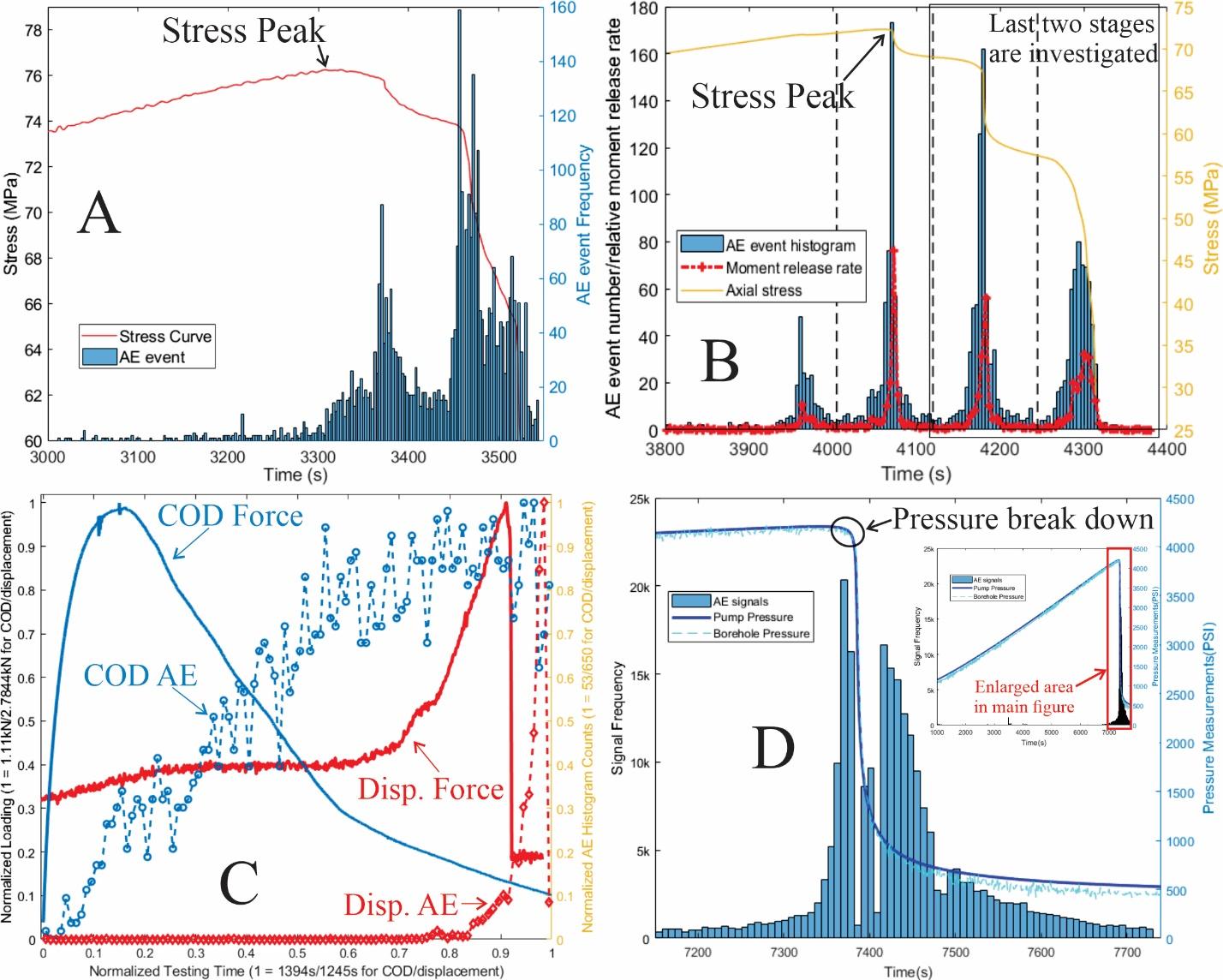


Fig. S16. Stress curves and AE histograms for: A) extending shear rock fracture^3^, B) confined shear rock fracture^4^, C) 3- or 4-point bending rock fracture^6,7^ using crack opening displacement (COD) loading and displacement loading, and D) hydraulic fracture^8,9^

#### Closed space shear:

Closed space shear rock fractures were from Ref.^4^. The specimen was of similar size to the extending shear one, and their loading protocols were similar as well. The shear rock fracture was confined by two coplanar open flaws having 60-degree inclination (Fig. 4c). The rock bridge between the two coplanar open flaws was approximately 25 mm, which is smaller than the specimen thickness. The energy releases under investigation were all within the post stress-peak loading domain (Fig. S15b). Its energy input is progressively decreasing as well. Again, to exclude or not to exclude the energy releases before the stress-peak will not alter the observations of significant clustering or non-clustering.

#### 3- or 4-point bending:

Tensile stress rock fractures were conducted at two different institutes by different groups of experimentalists. The tensile stress rock fractures were initiated from the notch cut at the center of the bending beams (Fig. 4d). One group used Crack Open Distance (COD) guide for the post stress-peak loading. The other group loaded the specimen to failure with monotonically increasing stress with small duration of post stress-peak (Fig. S15c). As such, the energy releases of one data set were obtained under a progressively decreasing energy input into the rock fracture, while the other was obtained primarily under a monotonically increasing energy input. This difference, as well as the difference in the data acquisition systems and sensors, has not altered the observations of non-clustering (global) or significant clustering (constrained to the specimens’ characterization lengths).

#### Hydraulic fracturing:

The case of hydraulic fracturing was from Ref.^9^. The specimen was approximately 250 mm × 150 mm × 150 mm. An injection well was drilled along the longitudinal axis (i.e., the 250 mm axis) into the center of the specimen. An inclined fault was pre-cut into the specimen, and this fault is some distance away from the bottom of injection well (Fig. 4e). During hydraulic fracturing, the borehole pressure can monotonically increase to the breakdown point, and later decreases progressively (Fig. S15d). It introduces two groups of temporally well-separated energy releases. One is prior to the breakdown, and the other is after. However, again, using all energy releases, prior breakdown energy releases, and post breakdown energy releases for the investigations will not alter the observations of non-clustering (global) or significant clustering (constrained).

#### Artificial rock-like specimen:

A test on the artificial rock material with ideal macroscale homogeneity and different loading protocols is investigated for further illustrating the magnitude clustering phenomenon is influenced by shear or tensile stress, and not influenced by the loading protocol. Artificial rock-like specimens were from Ref.^10^. The specimen configuration was similar to the extending shear one with slight difference on the height × width × depth. It also contains one central flaw. But this flaw was closed. The specimen was uniaxially loaded along the longitudinal direction using monotonically increasing stress. The source locations and the video records have cross validated that, there are two types of fractures that can be initiated during the test (Fig. S16a). One is tensile, from the loading range from 0% - 98% axial force (Fig. S16b); the other is shear, from primarily the loading range from 98% axial force to failure. This separation is approximate, however it is safe for extracting the stage of developing the rock fractures under tensile stress. Again, we observe a non-clustering (global) of energy releases for the stage that the developing rock fractures were under tensile stress. Significant clustering can be observed once we condition the inter-event distance to the level approximating the specimen thickness; or once we investigate all energy releases (0% axial force to failure) without applying any spatial condition, or investigate the energy releases for the stage that the developing rock fractures were under shear stressing without applying spatial condition.


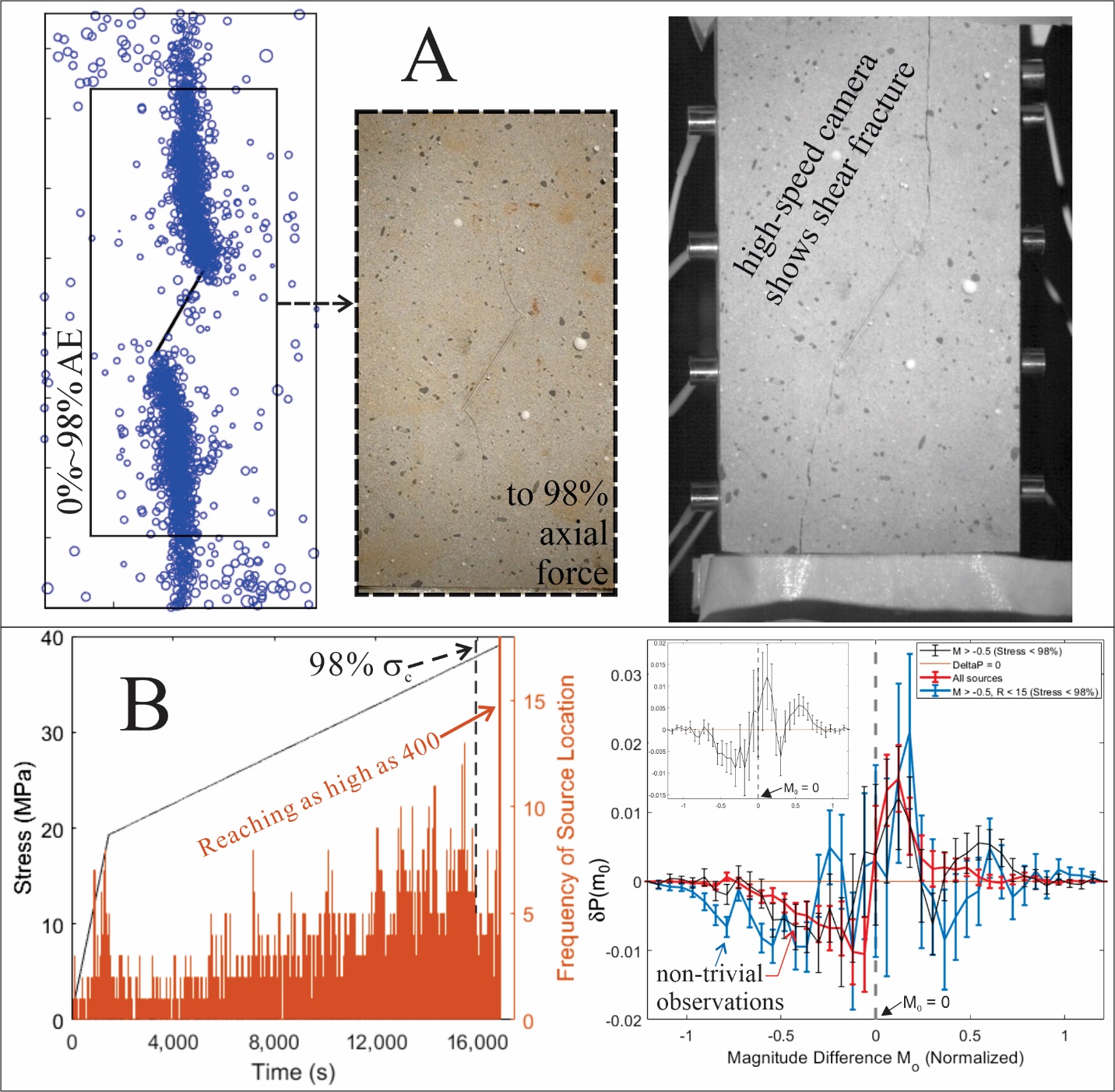


Fig. S17. A) induced fractures under tensile stress (left, to 98% axial force) and shear (right, under high-speed camera). B) Loading curve and AE event histogram (left), and the magnitude clustering observations (right) for sources < 98% axial force, all sources, and conditioned to R = 15 mm observation (thickness = 20 mm) for sources < 98% axial force. Error bars of 1 standard deviation are used.

### II-10. Influence of Rock Fracture Evolution

As shown in Fig. S13a, dropping the early-stage energy releases from the observation will enhance the significance of clustering. This observation holds true for investigations on both regional or global energy releases.

### II-11. Data Analysis Bridge

As shown in Fig. S14b, change of magnitude estimation methods will not alter the observation of significant clustering or non-clustering. Using first peak amplitudes of signals for the estimation has the best accuracy as in-situ sensor calibration has been conducted for the tests in Ref. ^3,4^. Thus, the magnitude clustering is of greater significance (labelled as “extending shear, First Peak, M > -0.6” in Fig. S14b). To note, the different labeling values, i.e. M > -0.6 for first peak estimation or M > -0.1 for overall peak estimation in Fig. S14b are reflecting the different magnitudes of completeness out of those estimations. Such difference is introduced by the fact that overall peak is of greater amplitude than the first peak of a signal.

Due to the different data acquisition systems and settings, the first peaks under some circumstances can be of greater uncertainty than the overall amplitude of signals, i.e. sampling rate is limited in Ref. ^9^. To ensure data from different systems can be analyzed and discussed together, we remove the calibration coefficients and re-do the analysis using overall peak amplitudes of the signals (see the two examples labelled as “Extending Shear, Overall Amplitude, …” in Fig. S14b). When in-situ sensor calibration information is available, the first peak is of greater accuracy than the overall peak. As expected, the analysis using overall peak amplitudes gives smaller statistical significance. However, the observation of significant clustering or non-clustering will not be altered by using different magnitude estimation methods. To be fair, the observation presented in Fig S14a are all based on overall amplitude estimation.

### II-12. Model seeks the violation of the null hypothesis of the Gutenberg-Richter law

The model building up the interevent triggering-triggered connection seeks the violation of the null hypothesis of Gutenberg-Richter law. It calculates the nearest neighbor in the normalized space-and-time domain. To find the trigger, if any, of a given AE event *j*, we calculate an event-pair metric of spatial and temporal correlation:

$n_{kj}=l_{kj}^{*}\times\tau_{kj}^{*}\equiv\left( r_{kj}^{Df}\times{10}^{-\frac{bm_{k}}{2}} \right)\times\left( t_{kj}\times{10}^{-\frac{bm_{k}}{2}} \right)$ (2)

for each of its preceding *k* events, where $l_{kj}^{*}$ and $\tau_{kj}^{*}$ are the normalized spatial and time distances between events *k* and *j*; $m_{k}$ is event *k*’s relative magnitude of AE moment; $Df$, *b* are the correlation fractal dimension of AEs, and b-value of the Gutenberg-Richter (G-R) law, respectively, which are directly estimated from the statistical features of AEs per test. The *i-*th event that satisfies $n_{ij}^{*}=n_{kj}$ is the most probable trigger of *j*-th event. A very low value of $n_{ij}^{*}$ indicates the existence of real triggering-triggered connection. A threshold of $n_{thresh}$ can be determined by observing the distribution of shuffled AE data in the normalized space-time plane^12^.

## Supplementary Note 3. Supplementary Table for the Statistical Significance of Magnitude Clustering of Field, Laboratory, and Synthetic Catalogs

Table S1. Table of statistical significance of each catalog's empirical cumulative density function (ECDF) pattern relative to a randomly shuffled version of the catalog.

| **Catalog** | **Number of S.D.** | **Confidence Level** |
| --- | --- | --- |
| *Field Catalogs* | | |
| California | 20.8 | >99.9999 |
| Hamilton (OH) | 5.6 | >99.9999 |
| Ryser (OH) | 3.2 | >99.9999 |
| Guthrie (OK) | 6.2 | >99.9999 |
| West Texas | 4.5 | 99.9996 |
| *Laboratory Catalogs* | | |
| Confined Shear | 8.0 | >99.9999 |
| Extending Shear | 16.8 | >99.9999 |
| Mixed Mode | 3.3 | 99.99 |
| *Synthetic Catalogs* | | |
| Stochastic | 0.3 | 25.7 |
| Stochastic Incomplete | 0.8 | 35.9 |
| Stochastic (4000 events) | 0.8 | 13.4 |
| Stochastic Smeared | 1.4 | 34.8 |
| ETAS | 1.0 | 26.9 |
| ETAS (4000 events) | 1.2 | 7.0 |
| ETAS.bayesian | 1.1 | 44.8 |
| ETAS.bayesian (Hamilton) | 1.0 | 58.9 |
| ETAS.bayesian (California) | 1.1 | 49.5 |
| ETAS (+22% repeating events) | 23.7 | >99.9999 |

Supplementary References:

1 Mizrahi, L., Nandan, S. & Wiemer, S. Embracing Data Incompleteness for Better Earthquake Forecasting. Journal of Geophysical Research: Solid Earth 126, doi:10.1029/2021jb022379 (2021).

2 Zhuang, J. & Touati, S. Stochastic simulation of earthquake catalogs. Community Online Resource for Statistical Seismicity Analysis, doi:doi:10.5078/corssa-43806322. (2015).

3 Xiong, Q. & Hampton, J. C. Non-local triggering in rock fracture. Journal of Geophysical Research: Solid Earth 125, doi:<https://doi.org/10.1029/2020JB020403> (2020).

4 Xiong, Q., Lin, Q. & Hampton, J. C. Temporal evolution of a shear-type rock fracture process zone (FPZ) along continuous, sequential, and spontaneous well-separated laboratory instabilities-from intact rock to thick gouged fault. Geophysical Journal International 226, 351–367, doi:<https://doi.org/10.1093/gji/ggab041> (2021).

5 Davidsen, J. & Green, A. Are earthquake magnitudes clustered? Phys Rev Lett 106, 108502, doi:10.1103/PhysRevLett.106.108502 (2011).

6 Lin, Q., Wan, B., Wang, S., Li, S. & Fakhimi, A. Visual Detection of a Cohesionless Crack in Rock under Three-Point Bending. Engineering Fracture Mechanics 211, 17-31, doi:<https://doi.org/10.1016/j.engfracmech.2019.02.009> (2019).

7 Lin, Q., Wan, B., Wang, Y., Lu, Y. & Labuz, J. F. Unifying acoustic emission and digital imaging observations of quasi-brittle fracture. Theoretical and Applied Fracture Mechanics 103, 102301, doi:10.1016/j.tafmec.2019.102301 (2019).

8 Xiong, Q. & Hampton, J. C. A Laboratory Observation on the Acoustic Emission Point Cloud Caused by Hydraulic Fracturing, and the Post-pressure Breakdown Hydraulic Fracturing Re-activation due to Nearby Fault. Rock Mechanics and Rock Engineering 54, 5973–5992, doi:<https://doi.org/10.1007/s00603-021-02585-x> (2021).

9 Hampton, J., Gutierrez, M. & Matzar, L. Microcrack Damage Observations near Coalesced Fractures Using Acoustic Emission. Rock Mechanics and Rock Engineering 52, 3597-3608, doi:10.1007/s00603-019-01818-4 (2019).

10 Pan, X., Xiong, Q. & Wu, Z. New Method for Obtaining the Homogeneity Index m of Weibull Distribution Using Peak and Crack-Damage Strains. International Journal of Geomechanics 18, doi:10.1061/(ASCE)GM.1943-5622.0001146 (2018).

11 Maghsoudi, S., Eaton, D. W. & Davidsen, J. Nontrivial clustering of microseismicity induced by hydraulic fracturing. Geophysical Research Letters 43, 10,672-610,679, doi:10.1002/2016gl070983 (2016).

12 Davidsen, J. et al. Triggering Processes in Rock Fracture. Phys Rev Lett 119, 068501, doi:10.1103/PhysRevLett.119.068501 (2017).
